# Regulation of circadian clock transcriptional output by CLOCK:BMAL1

**DOI:** 10.1371/journal.pgen.1007156

**Published:** 2018-01-04

**Authors:** Alexandra J. Trott, Jerome S. Menet

**Affiliations:** Department of Biology, Program of Genetics and Center for Biological Clocks Research, Texas A&M University, College Station, TX, United States of America; Charité - Universitätsmedizin Berlin, GERMANY

## Abstract

The mammalian circadian clock relies on the transcription factor CLOCK:BMAL1 to coordinate the rhythmic expression of 15% of the transcriptome and control the daily regulation of biological functions. The recent characterization of CLOCK:BMAL1 cistrome revealed that although CLOCK:BMAL1 binds synchronously to all of its target genes, its transcriptional output is highly heterogeneous. By performing a meta-analysis of several independent genome-wide datasets, we found that the binding of other transcription factors at CLOCK:BMAL1 enhancers likely contribute to the heterogeneity of CLOCK:BMAL1 transcriptional output. While CLOCK:BMAL1 rhythmic DNA binding promotes rhythmic nucleosome removal, it is not sufficient to generate transcriptionally active enhancers as assessed by H3K27ac signal, RNA Polymerase II recruitment, and eRNA expression. Instead, the transcriptional activity of CLOCK:BMAL1 enhancers appears to rely on the activity of ubiquitously expressed transcription factors, and not tissue-specific transcription factors, recruited at nearby binding sites. The contribution of other transcription factors is exemplified by how fasting, which effects several transcription factors but not CLOCK:BMAL1, either decreases or increases the amplitude of many rhythmically expressed CLOCK:BMAL1 target genes. Together, our analysis suggests that CLOCK:BMAL1 promotes a transcriptionally permissive chromatin landscape that primes its target genes for transcription activation rather than directly activating transcription, and provides a new framework to explain how environmental or pathological conditions can reprogram the rhythmic expression of clock-controlled genes.

## Author summary

Circadian clocks in mammals rely on the heterodimeric transcription factor CLOCK:BMAL1 to drive rhythmic gene expression and allow biological functions to perform best at the most appropriate time of the day. Investigation of the mechanisms by which CLOCK:BMAL1 regulates its target genes transcription has led to the paradoxical observation that while CLOCK:BMAL1 DNA binding is rhythmic and occurs during the day for all target genes, its transcriptional output is highly heterogeneous. To address this issue, we analyzed independent genome-wide datasets and found that CLOCK:BMAL1 DNA binding during the day is associated with a reorganization of the chromatin that is favorable to transcription activation for all target genes. However, this diurnal CLOCK:BMAL1 DNA binding and chromatin remodeling is not sufficient to promote a transcriptionally active enhancer, therefore suggesting that CLOCK:BMAL1 cooperates with other factors to control the transcription of most of its target genes. This hypothesis is supported by our finding that ubiquitous transcription factors, but not tissue-specific transcription factors, are differentially recruited at CLOCK:BMAL1 enhancers. Altogether, our data highlight the critical role of transcription factors recruited at CLOCK:BMAL1 enhancers in regulating transcription, and present a new mechanistic framework to understand how changes in the environment can reprogram circadian transcriptional programs.

## Introduction

Virtually every mammalian cell harbors a circadian clock that regulates rhythmic gene expression to enable biological functions to occur at the most appropriate time of day. Circadian clocks rely on transcriptional feedback loops which are initiated in mammals by the heterodimeric transcription factor CLOCK:BMAL1 [for review, 1]. CLOCK:BMAL1 rhythmically binds to DNA to activate the rhythmic transcription of the core clock genes *Period* (*Per1*, *Per2*, *Per3*), *Cryptochrome* (*Cry1* and *Cry2*), *Rev-erb* (*Rev-erbα* and *Rev-erbβ*) and *Ror* (*Rorα*, *Rorβ* and *Rorγ*). Upon expression and maturation, PERs and CRYs form a repressive complex that rhythmically inhibits CLOCK:BMAL1-mediated transcription first on-DNA and then off-DNA [2–5]. Furthermore, REV-ERBs and RORs rhythmically regulate *Bmal1* expression by repressing or activating its transcription, which promotes robustness of circadian oscillations [6, 7]. In addition to activating the rhythmic transcription of core clock components, CLOCK:BMAL1 also regulates rhythmic expression of thousands of clock-controlled genes to generate oscillations in biochemistry, physiology and behavior, and thus control the rhythmic organization of most biological functions [8–10].

Characterizing the mechanisms through which CLOCK:BMAL1 regulates expression of its target genes has largely been carried out by determining how CLOCK:BMAL1 regulates the transcription of core clock genes (*Per*, *Cry* and *Rev-erb*), and target genes (e.g., *Dbp*, named for *D site of albumin promoter binding protein*). Results from many laboratories show that the rhythmic binding of CLOCK:BMAL1 to e-boxes located in core clock gene promoters is necessary and sufficient for rhythmic transcription [4, 5, 11–13]. Upon DNA binding during the light phase, CLOCK:BMAL1 promotes chromatin modifications by recruiting histone-modifying enzymes to core clock gene promoters and enhancers. These enzymes include the histone acetyltransferases p300 and CBP, which mediate the acetylation of H3K9 and H3K27, and the histone methyltransferases MLL1 and MLL3 (Myeloid/Lymphoid Or Mixed-Lineage Leukemia 1 and 3), which promote the tri-methylation of H3K4 [3, 14–19]. CLOCK:BMAL1 rhythmic DNA binding was also recently shown to promote rhythmic nucleosome removal, thereby generating a chromatin landscape that is favorable for the binding of other transcription factors at its enhancers [20]. Finally, CLOCK:BMAL1 recruits transcriptional co-activators, including components of the mediator complex and RNA Polymerase II (Pol II) to initiate core clock gene transcription [3, 21, 22]. During the repression phase in the early night, binding of the PER/CRY complex to DNA-bound CLOCK:BMAL1 is accompanied by the co-recruitment of histone deacetylases and demethylases and the removal of the H3K9ac, H3K27ac and H3K4me3 marks [19, 23–27]. While these mechanisms are required for CLOCK:BMAL1-mediated transcription activation of core clock genes, it still remains unclear if the same mechanisms regulate the rhythmic expression of clock-controlled genes.

The recent characterization of CLOCK and BMAL1 mouse liver cistromes revealed that although CLOCK:BMAL1 binds synchronously during the middle of the day to thousands of enhancers and promoters, the transcription of its target genes is highly heterogeneous [3, 28–30]. Indeed, not all CLOCK:BMAL1 target genes are rhythmically expressed, and a large fraction of the rhythmically expressed target genes are transcribed at night, in antiphase to maximal CLOCK:BMAL1 DNA binding [28]. These data therefore suggest that the mechanisms by which CLOCK:BMAL1 regulates transcription of core clock genes differs from the regulation of other clock-controlled genes, and that additional mechanisms account for the activation of rhythmic gene expression by the circadian clock.

To uncover these mechanisms and to delineate the transcriptional logic underlying CLOCK:BMAL1 heterogeneous transcriptional output, we performed a meta-analysis of genome-wide datasets investigating the molecular events occurring at CLOCK:BMAL1 DNA binding sites, including CLOCK:BMAL1 rhythmic DNA binding, epigenetic modifications and transcription activation. Our analysis reveals that while CLOCK:BMAL1 DNA binding is sufficient to decondense the chromatin and prime its enhancers for transcriptional activation, it is not sufficient to generate transcriptionally active enhancers. Our results also indicate that many transcription factors bind to CLOCK:BMAL1 enhancers, and their recruitment likely contributes to CLOCK:BMAL1 clock-controlled transcriptional output. Altogether, our data support that CLOCK:BMAL1 regulation of clock-controlled gene expression relies on the cooperation between CLOCK:BMAL1 and other transcription factors. Furthermore, our data also suggest that a major role of CLOCK:BMAL1 is to generate a permissive chromatin landscape to rhythmically prime its enhancers for the recruitment of other transcription factors, rather than directly promoting transcription activation.

## Results

### CLOCK:BMAL1 transcriptional output is heterogeneous

**Fig 1:**
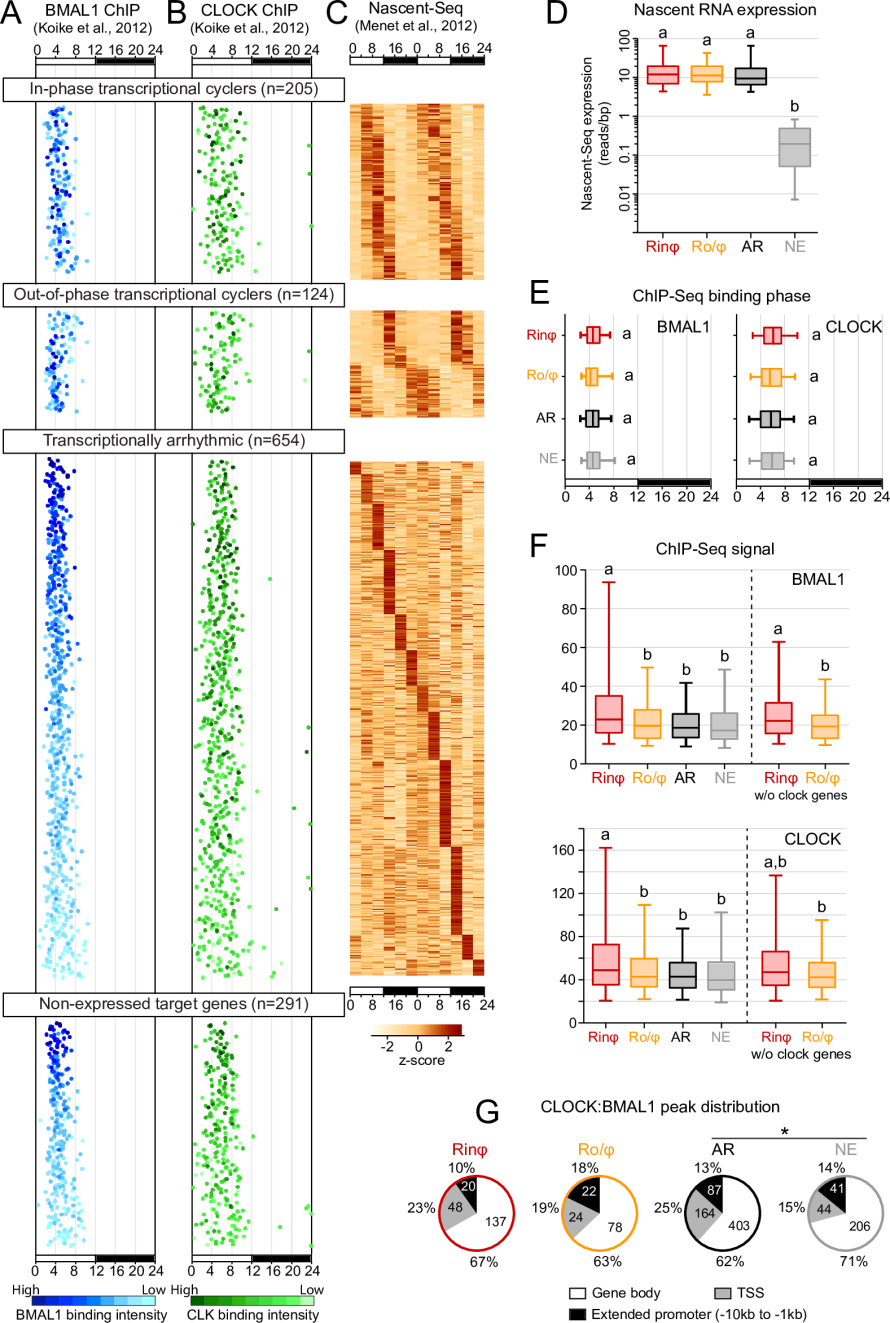
Mouse liver CLOCK:BMAL1 transcriptional output is heterogeneous. A, B. Mouse liver BMAL1 (blue, A) and CLOCK (green, B) ChIP-Seq peaks from Koike et al., 2012 were mapped to their target genes and parsed based on their transcriptional output (Nascent-Seq from Menet et al., 2012, C). Each dot represents the phase of maximal DNA binding, and the ChIP-Seq signal is displayed using different shades of color to illustrate differences in binding intensity. C. Heatmap representation of the Nascent-Seq signal of direct CLOCK:BMAL1 target genes classified based on their transcriptional output in the mouse liver. Each lane represents the Nascent-Seq signal of a gene corresponding to the CLOCK and BMAL1 peaks in A and B. Nascent-Seq signal was ordered based on the phase of nascent RNA oscillations for the in-phase and out-of-phase transcriptional cyclers. Ordering of arrhythmically transcribed genes is based on the peak time of maximal expression; the lack of a distinctive 24-hr rhythm profile of nascent RNA expression over the 48-hr time-scale is indicative of arrhythmic transcription. No heatmap could be generated for the non-expressed genes because of the lack of nascent RNA expression. D. Nascent RNA expression, calculated as reads/bp, for each of the 4 CLOCK:BMAL1 transcriptional output group (Rinφ: in-phase transcriptional cyclers; Ro/φ: out-of-phase transcriptional cyclers; AR: arrhythmically transcribed target genes; NE: non-expressed target genes). Groups with different letters are significantly different (Kruskal-Wallis test; p < 0.05). E. Phase of maximal BMAL1 (left) and CLOCK (right) rhythmic DNA binding for each of the 4 CLOCK:BMAL1 transcriptional output categories. Groups with different letters are significantly different (Kruskal-Wallis test; p < 0.05). F. BMAL1 (top) and CLOCK (bottom) ChIP-Seq signal for each of the 4 CLOCK:BMAL1 transcriptional output groups. Signal is also displayed for the Rinφ and Ro/φ groups after removal of the ChIP-Seq signal at peaks targeting core clock genes. Groups with different letters are significantly different (Kruskal-Wallis test; p < 0.05). G. Location of CLOCK:BMAL1 peaks within gene loci for each of the 4 CLOCK:BMAL1 transcriptional output groups. TSS (transcriptional start site; +/- 1kb from annotated TSS); Gene body: + 1kb from TSS to + 1kb from transcription termination site; Extended promoter: - 10 kb to—1 kb from the annotated TSS. Numbers correspond to the percentage and numbers of peaks (outside and inside the pie chart, respectively) within each location for each group. * denotes a significant difference in the distribution of peaks between the AR and NE groups (chi square test; p < 0.05).

To characterize the mechanisms by which CLOCK:BMAL1 regulates the transcriptional activity of its target genes at the genome-wide level in the mouse liver, we first generated a list of high-confidence CLOCK:BMAL1 DNA binding sites by determining the overlap between CLOCK and BMAL1 ChIP-Seq peaks in the mouse liver [3]. This analysis resulted in a list of 3217 CLOCK:BMAL1 binding sites, of which 2458 peaks can be assigned to a direct target gene (i.e., a CLOCK:BMAL1 peak located between -10kb of a target gene transcription start site and +1kb of a target gene transcription termination site; see S1 Fig, S1 Table, and methods section for details). To determine the extent to which rhythmic CLOCK:BMAL1 DNA binding contributes to rhythmic transcription activation at the genome-wide level, we used a public mouse liver Nascent-Seq dataset that characterized the levels of nascent RNA expression over the course of a 24-hr day [28]. A Nascent-Seq dataset was preferred over RNA-Seq because nascent RNA expression directly reflects transcription activation, and is unaffected by the post-transcriptional regulations that contribute to rhythmic mRNA expression in the mouse liver [3, 28, 31]. We found that only a small fraction of CLOCK:BMAL1 target genes are rhythmically transcribed (~26%; S1 Fig). Noticeably, not all rhythmic target genes are transcribed during the day, i.e., coincidently with rhythmic CLOCK:BMAL1 DNA binding (ZT02-ZT12). Indeed, 38% of the rhythmic CLOCK:BMAL1 target genes exhibit a peak of transcription between ZT12 and ZT02, out-of-phase with the rhythmic DNA binding of CLOCK:BMAL1 (n = 124 CLOCK:BMAL1 peaks) (Fig 1A–1C; S1 Fig). Importantly, our analysis also reveals that the majority of CLOCK:BMAL1 direct target genes are either arrhythmically transcribed (AR; n = 654 CLOCK:BMAL1 peaks) or not expressed (NE; n = 291 CLOCK:BMAL1 peaks) (Fig 1A–1D; S1 Fig). To determine if this result may be due to comparing samples collected in constant darkness (ChIP-Seq) and in a light:dark (LD) cycle (Nascent-Seq), we also analyzed a mouse liver BMAL1 ChIP-Seq rhythm performed under LD condition [29]. BMAL1 binding phase and ChIP-Seq signal under LD condition both exhibit a remarkably high level of similarity to those under DD conditions, and this even for the AR or NE target genes (S2 Fig). This therefore suggests that the large number of arrhythmically transcribed or not expressed CLOCK:BMAL1 target genes is not a consequence of using datasets generated under different lighting conditions. Taken together, these results indicate that the mechanisms underlying CLOCK:BMAL1-mediated rhythmic transcription of core clock genes (i.e., *Per1*, *Per2*, *Per3*, *Cry1*, *Cry2*, *Rev-erbα*, *Rev-erbβ* and *Dbp*) are not prevalent at the genome-wide level. They also suggest that the rhythmic recruitment of CLOCK:BMAL1 at its target gene promoters and enhancers is not sufficient to activate transcription for the majority of its target genes.

### CLOCK:BMAL1 heterogeneous transcriptional output is not mediated by differences in CLOCK:BMAL1 DNA binding

To investigate the mechanisms underlying CLOCK:BMAL1 heterogeneous transcriptional output, we first examined if differences in the phase, intensity or location of CLOCK:BMAL1 DNA binding might explain the differences in transcription activation. The phase of CLOCK:BMAL1 DNA binding was found to be indistinguishable between all four transcriptional output categories, as both CLOCK and BMAL1 rhythmically bind to DNA with a peak between ZT3 and ZT9 for almost all target genes (Fig 1A, 1B and 1E). We then used CLOCK and BMAL1 ChIP-Seq signal as a readout to determine DNA binding intensity, and found that both CLOCK and BMAL1 ChIP-Seq signals are significantly higher at DNA binding sites targeting the in-phase transcriptional cyclers (Rinφ) when compared to peaks targeting the 3 other groups (out-of phase cyclers, arrhythmically expressed and non-expressed target genes) (Fig 1A, 1B and 1F; Kruskal-Wallis test, p < 0.05). Remarkably, the binding intensity of CLOCK and BMAL1 at non-expressed target genes (NE) is similar to the binding intensity observed at the out-of-phase transcriptional cyclers (Ro/φ) and arrhythmically transcribed (AR) target genes, suggesting that CLOCK:BMAL1 DNA binding alone does not directly activate transcription at most of its target genes (e.g., comparisons between Fig 1D and 1F). To verify that these results are not due to the cut-offs we used to partition CLOCK:BMAL1 transcriptional output, we performed similar analyses using direct correlations between BMAL1 or CLOCK ChIP-Seq signal and the phase of rhythmic transcription, as well as by partitioning rhythmic target genes in five groups of equal sizes. These analyses confirmed our results (S3 and S4 Figs). While rhythmically transcribed target genes peaking from ZT5 to ZT13 exhibit higher BMAL1 and CLOCK ChIP-Seq signal, no differences in DNA binding signal were observed between the rhythmically expressed targets peaking from ZT13 to ZT5 and the AR and NE groups (S3 Fig). In addition, we did not find any significant correlation between either CLOCK or BMAL1 ChIP-Seq signals and the phase of DNA binding or the phase of rhythmic transcription (S4 Fig). Because CLOCK:BMAL1 peaks targeting core clock genes are enriched in the Rinφ and Ro/φ groups and exhibit higher ChIP-Seq signal than clock-controlled (output) genes, we also compared CLOCK and BMAL1 ChIP-Seq signals between groups after removing peaks targeting the core clock genes (i.e., comparing clock-controlled genes only). Whereas BMAL1 ChIP-Seq signal intensity was still significantly higher at the Rinφ target genes compared to the three other groups, CLOCK DNA binding intensity was similar between all 4 groups (Fig 1F). Our data therefore indicate that while higher BMAL1 DNA binding signal may contribute to Rinφ transcription, the different transcriptional output of CLOCK:BMAL1 target genes cannot be explained solely by differences in CLOCK:BMAL1 DNA binding intensity.

We also examined if differences in the location of CLOCK:BMAL1 DNA binding sites are associated with differences in transcriptional output by mapping CLOCK:BMAL1 peaks to either the transcription start site (TSS), gene body or extended promoter (-10 kb to -1 kb from the TSS) of their target genes. While the AR and NE groups were found to be statistically different (chi square test; p < 0.05), we did not observe any differences between the rhythmic target groups (Rinφ and Ro/φ) and the arrhythmically or not expressed groups (Fig 1G). The vast majority of CLOCK:BMAL1 peaks were located within enhancers (i.e., gene body or extended promoter), and only ~10–19% of CLOCK:BMAL1 peaks were mapped to TSS. Finally, we examined if differences in the number of genes targeted by multiple CLOCK:BMAL1 peaks were associated with differences in transcriptional output. We found that in-phase transcriptional cyclers were more frequently targeted by multiple CLOCK:BMAL1 peaks, and that conversely, non-expressed target genes were less frequently targeted by multiple peaks (S5 Fig). However, the lack of differences between the Ro/φ and AR groups indicates that the presence of multiple ChIP-Seq peaks does not directly influence the rhythmicity of CLOCK:BMAL1 target genes.

Taken together, our analysis indicates that CLOCK:BMAL1 heterogeneous transcriptional output can not be simply attributed to differences in the phase, intensity or location of CLOCK and BMAL1 binding to the DNA. While stronger DNA binding intensity may contribute to rhythmic transcription during the light phase, additional mechanisms are likely to contribute to CLOCK:BMAL1 transcriptional output heterogeneity.

### Recruitment of PERs and CRYs at CLOCK:BMAL1 DNA binding sites does not contribute to the heterogeneous CLOCK:BMAL1 transcriptional output

Circadian repression in mammals is initiated at the beginning of the night by the recruitment of the PER/CRY repressive complex and its associated histone deacetylases and methyltransferases to CLOCK:BMAL1 on DNA [23–25, 27, 32]. Because a differential recruitment of PERs and CRYs at CLOCK:BMAL1 DNA binding sites could lead to differences in CLOCK:BMAL1-mediated transcriptional output (e.g., decreased recruitment at arrhythmically transcribed target genes, delayed recruitment of out-of-phase transcriptional cyclers, *etc*.), we investigated the DNA binding profile of PER1, PER2, CRY1 and CRY2 at CLOCK:BMAL1 DNA binding sites for each of the four transcriptional output groups using publically available ChIP-Seq datasets [3].

Our analysis shows that PER1, PER2 and CRY2 are rhythmically recruited at CLOCK:BMAL1 DNA binding sites with little difference between the four transcriptional output groups (S6 Fig). Maximal DNA binding for PER1, PER2 and CRY2 occur at CT12-16 for all groups, and differences were mostly observed for CRY2, where higher ChIP-Seq signal was found for rhythmically expressed target genes (S6 Fig). On the other hand, analysis of CRY1 recruitment to CLOCK:BMAL1-bound enhancers revealed more pronounced differences between all four groups. CRY1 is a potent circadian repressor that is preferentially recruited at the beginning of the light phase just prior CLOCK:BMAL1 transcription activation (i.e., CT0-4), a mechanism proposed to poise CLOCK:BMAL1 for transcription activation [3]. We found that CRY1 recruitment at CT4 is significantly higher for rhythmically transcribed target genes (both Rinφ and Ro/φ) than for arrhythmically transcribed and non-expressed genes (S6 Fig). In addition, CRY1 recruitment was significantly decreased in non-expressed CLOCK:BMAL1 target genes than arrhythmic genes at CT4. These data thus suggest that CRY1 recruitment to CLOCK:BMAL1 DNA binding sites is, in addition to its well-characterized repressive effect, linked to rhythmic transcription activation. Consistent with this hypothesis are the higher levels for Ro/φ at CT12 compared to Rinφ (S6 Fig).

### REV-ERBα and REV-ERBβ ChIP-Seq signal is higher at CLOCK:BMAL1 peaks targeting genes transcribed at night

**Fig 2:**
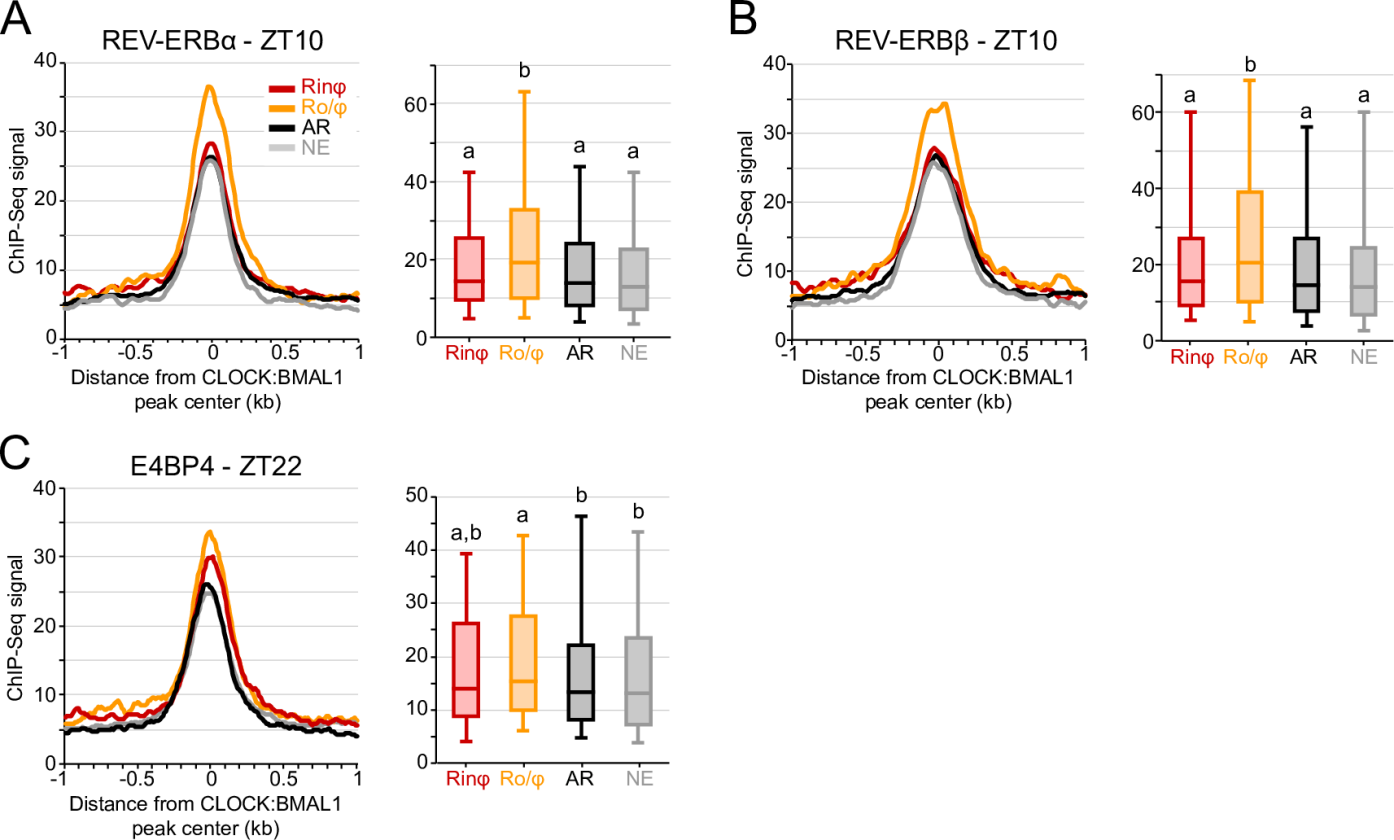
REV-ERBα and REV-ERBβ ChIP-Seq signal is higher at CLOCK:BMAL1 DNA binding sites targeting genes transcribed at night. A-C *(Left)*. Average ChIP-Seq signal for REV-ERBα (A), REV-ERBβ (B) and E4BP4 (C) at CLOCK:BMAL1 DNA binding sites (center ± 1kb) for each of the 4 CLOCK:BMAL1 transcriptional output group. A-C (*Right)*. Distribution of REV-ERBα (A), REV-ERBβ (B) and E4BP4 (C) ChIP-Seq signal at CLOCK:BMAL1 peaks for each of the 4 CLOCK:BMAL1 transcriptional output groups (signal for each peak was averaged at CLOCK:BMAL1 peak center ± 250bp). Groups are labeled as in Fig 1. Those with different letters are significantly different (Kruskal-Wallis test; p < 0.05). REV-ERBα and REV-ERBβ ChIP were performed from mice liver collected at ZT10, while E4BP4 ChIP was performed from mice liver collected at ZT22. ChIP-Seq datasets were retrieved from Cho et al., 2012 [35], and E4BP4 ChIP-Seq datasets from Fang et al., 2014 [36].

Based on the mechanisms mediating the delayed transcription of the CLOCK:BMAL1 target gene *Cry1* [33], a model incorporating the nuclear receptors *Rev-erb* (repressor) and *Ror* (activator), and the D-box transcriptional factors *E4bp4* (also called *Nfil3*; repressor), *Dbp*, *Hlf* and *Tef* (activators) has been proposed to explain the different phases of rhythmic gene expression in the mouse liver [33, 34]. In this model, co-binding of D-box transcription factors with CLOCK:BMAL1 is proposed to delay the phase of CLOCK:BMAL1 target genes from the morning to the afternoon (i.e., from ~ZT6 to ~ZT12), and additional binding of REV-ERBs and RORs would further delay the phase of transcription to the night (e.g., ~ZT18). To test if the binding of REV-ERBs and D-box transcription factors contribute to the delay of the out-of-phase CLOCK:BMAL1 target genes, we used publicly available ChIP-Seq datasets to determine REV-ERBα, REV-ERBβ [35], and E4BP4 [36] DNA binding intensity at CLOCK:BMAL1 enhancers. We find that REV-ERBα and REV-ERBβ DNA binding, which peaks at ZT10 for all target genes [35], is significantly higher at CLOCK:BMAL1 peaks targeting genes transcribed during the night [consistent with the model proposed based on Cry1 expression; 33, 34], and no differences were observed between Rinφ, AR and NE target genes (Fig 2A and 2B; Kruskal-Wallis test, p < 0.05). The binding of E4BP4, which is maximal at ZT22 [36], was also enriched at CLOCK:BMAL1 enhancers targeting the Ro/φ genes, but to a lesser extent than what was observed for the REV-ERBs (Fig 2C). In particular, no significant difference in enrichment was observed between the Rinφ and the Ro/φ groups, perhaps because co-binding of both CLOCK:BMAL1 and D-box transcription factors drives rhythmic transcription in the afternoon around ZT12, a time used for our cut-off to differentiate the in-phase from out-of-phase transcription cyclers. In summary, our analysis indicates that the binding of REV-ERBα and REV-ERBβ (and eventually E4BP4) at CLOCK:BMAL1 enhancers may, as suggested by others [33, 34], contribute to the delayed transcription of rhythmically expressed CLOCK:BMAL1 target genes.

### CLOCK:BMAL1 promotes rhythmic nucleosome removal independently of its transcriptional output

Our inability to detect substantial differences in CLOCK:BMAL1 DNA binding that would explain the heterogeneity of CLOCK:BMAL1 transcriptional output suggests that mechanisms other than the recruitment of core clock proteins to target gene promoters control CLOCK:BMAL1-mediated transcription. The recent finding that CLOCK:BMAL1 promotes the removal of nucleosomes when bound to DNA may represent one of these mechanisms [20]. Indeed, by mediating the removal of nucleosomes, CLOCK:BMAL1 would enable other transcription factors to access CLOCK:BMAL1 enhancers (most transcription factors bind better to naked DNA than DNA wrapped around nucleosomes).

**Fig 3:**
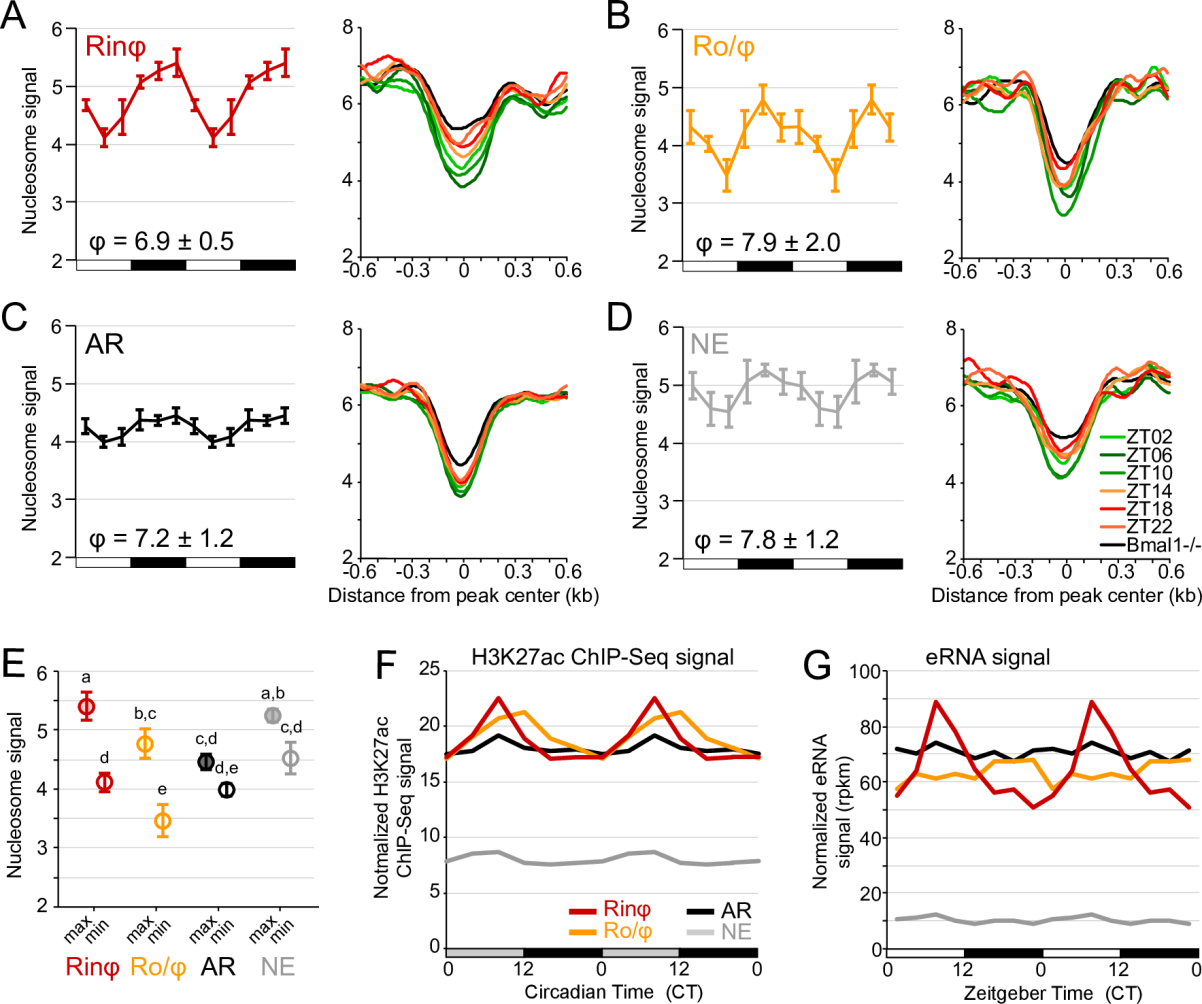
CLOCK:BMAL1 rhythmic DNA binding is associated with rhythmic nucleosome signal, but not with rhythmic histone post-translational modifications and eRNA transcription. A-D. Rhythmic nucleosome signal at CLOCK:BMAL1 DNA binding sites for each of the 4 CLOCK:BMAL1 transcriptional output groups: (A) Rhythmic-in-phase (Rinφ, red); (B) Rhythmic out-of-phase (Ro/φ, orange); (C) arrhythmic (AR, black); (D) non expressed (NE, grey) target genes). Nucleosome signal was retrieved from mouse liver MNase-Seq datasets [20], which consists of 6 time points each separated by 4 hours with n = 4 mice for each time point. (*Left*): 6-time points rhythm of nucleosome signal at CLOCK:BMAL1 binding sites (calculated at CLOCK:BMAL1 peak center ± 75 bp for each peak), displayed as the average ± s.e.m. of the signal (n = 4) calculated for each mouse and for each transcriptional output category. The phase of rhythm (average ± s.e.m. from 4 independent rhythm) is indicated in the bottom right. Each rhythm is double-plotted for better visualization. (*Right*): average nucleosome signal for each transcriptional output group at CLOCK:BMAL1 DNA-binding sites (±0.6 kb) during the light phase (ZT2, ZT6, and ZT10; green) and dark phase (ZT14, ZT18, and ZT22; red/orange) of wild-type mice and in Bmal1-/- mice (average signal for six time points; black). E. maximal and minimal nucleosome signal from the 6-time points rhythms for each of the CLOCK:BMAL1 transcriptional output groups. Groups with different letters are significantly different (2-way ANOVA; p < 0.05). F. Circadian rhythm of H3K27ac ChIP-Seq signal in the mouse liver at CLOCK:BMAL1 DNA binding sites (calculated at CLOCK:BMAL1 peak center ± 1 kb) for each of the 4 CLOCK:BMAL1 transcriptional output group. Datasets were retrieved from Koike et al., 2012 [3] and re-analyzed (see methods for more details). Values correspond to the ChIP-Seq signal median for each group. Each rhythm is double-plotted for better visualization. G. Rhythm of enhancer RNA (eRNA) signal in the mouse liver at CLOCK:BMAL1 DNA binding sites (calculated at CLOCK:BMAL1 peak center ± 500 bp) for each of the 4 CLOCK:BMAL1 transcriptional output groups. Datasets were retrieved from Fang et al., 2014 [36] and re-analyzed (see methods for more details). Values correspond to the eRNA signal median for each group. Each rhythm is double-plotted for better visualization.

To test if CLOCK:BMAL1-mediated nucleosome removal can contribute to the heterogeneity of CLOCK:BMAL1 transcriptional output, we examined mouse liver nucleosome signal over the 24-hr day at CLOCK:BMAL1 DNA binding sites for each of the transcriptional output groups, using a public MNase-Seq dataset (micrococcal nuclease digestion of mouse liver chromatin at 6-time points and high-throughput sequencing of mononucleosomes [20]). Our analysis reveals that nucleosome signal is rhythmic at CLOCK:BMAL1 DNA binding sites for each of the transcriptional output categories, i.e., even at CLOCK:BMAL1 DNA binding sites targeting arrhythmically transcribed and non-expressed genes (Fig 3A–3D; S7 Fig). Importantly, the phase of the rhythms is similar for all groups and minimal nucleosome signal coincides with maximal CLOCK:BMAL1 DNA binding during the light phase. Closer inspection of the levels of nucleosome signal and rhythm amplitude reveals important differences between each of the four transcriptional output categories (Fig 3E). First, the amplitude of the rhythms is significantly decreased for arrhythmically transcribed target genes. While minimal levels of nucleosome signal during the day are similar between the AR and Rinφ groups, nucleosome signal remains low during the night (i.e., when CLOCK:BMAL1 is not bound to DNA) at CLOCK:BMAL1 peaks targeting AR genes (Fig 3E). This suggests that some transcription factors may be still bound to DNA during the night in the AR group (when CLOCK:BMAL1 is not bound to DNA), thereby preventing the reformation of nucleosomes. This may promote transcription at night and thus lead to arrhythmic transcription. Second, the overall nucleosome signal is significantly lower at CLOCK:BMAL1 peaks targeting Ro/φ genes than for Rinφ genes, without any significant effect on the amplitude of the rhythm (Fig 3E). In addition, the time of minimal nucleosome signal is delayed by 4 hours between Rinφ and Ro/φ: while it coincides with the time of maximal CLOCK:BMAL1 DNA binding for Rinφ genes (ZT06), minimal nucleosome signal is observed at ZT10 for Ro/φ genes. This delayed nucleosome signal for the out-of-phase transcriptional cyclers may be explained by the significant recruitment of REV-ERBα and REV-ERBβ (Fig 2A and 2B). Indeed, CLOCK:BMAL1 has been recently proposed to facilitate circadian repression by promoting the recruitment of REV-ERBα through chromatin decondensation [37]. Thus, the increased binding of REV-ERBs at CLOCK:BMAL1 enhancers at ZT10 may promote a further decrease in nucleosome signal. Furthermore, anti-phase binding of the RORs on ROREs during the night would prevent a full nucleosome re-compaction, thereby promoting lower levels of nucleosome signal at CLOCK:BMAL1 peaks targeting Ro/φ target genes. Finally, there are no significant differences of nucleosome signal between CLOCK:BMAL1 DNA binding sites targeting in-phase transcriptional cyclers than those targeting non-expressed target genes (Fig 3E). This intriguing result suggests that although CLOCK:BMAL1 is unable to promote transcription activation at NE target genes, its rhythmic DNA binding still mediates a rhythm in nucleosome signal. One possible explanation for this result is that CLOCK:BMAL1 decondenses the chromatin to facilitate the binding of other transcription factors, but those would not be recruited at NE target genes except under specific conditions (e.g. environmental stressors), thereby preventing activation of transcription under standard conditions.

### CLOCK:BMAL1 does not directly contribute to the transcriptional activity of its enhancers

Our data indicate that CLOCK:BMAL1 rhythmic DNA binding promotes the rhythmic removal of nucleosomes at all four transcriptional output categories. We then asked if CLOCK:BMAL1 can also promote the formation of transcriptionally active enhancers. To address this question, we used public datasets [3, 36, 38] to examine the rhythmic pattern of two independent markers of enhancer activity at CLOCK:BMAL1 DNA binding sites: the post-translational modification H3K27 acetylation (H3K27ac), which positively correlates with enhancer activity at almost all enhancers and TSS [39], and the expression levels of enhancer RNA (eRNA), which are relatively short non-coding RNA molecules (50–2000 nucleotides) transcribed at active enhancer regions [40].

While mouse liver H3K27ac ChIP-Seq signal is rhythmic and high during the light phase at CLOCK:BMAL1 DNA binding sites targeting in-phase transcriptional cyclers (consistent with CLOCK:BMAL1 directly facilitating the acetylation of H3K27; Fig 3F and S8 Fig), significant differences were observed at CLOCK:BMAL1 DNA binding sites targeting the other 3 transcriptional output categories. Rhythmic H3K27ac rhythm is delayed for the out-of-phase transcriptional cyclers, and the amplitude of H3K27ac rhythm is significantly dampened at CLOCK:BMAL1 DNA binding sites targeting arrhythmically transcribed genes (Fig 3F and S8A Fig). Remarkably, levels of H3K27ac are close to background levels at CLOCK:BMAL1 peaks targeting non-expressed genes. Given that CLOCK:BMAL1 rhythmically binds to relatively similar levels for all four transcriptional output categories, our analysis suggests that CLOCK:BMAL1 DNA binding does not directly contribute to the acetylation of H3K27.

To extend on this observation, we then examined another marker of enhancer transcriptional activity by assessing eRNA expression at CLOCK:BMAL1 DNA binding sites using a publicly available GRO-Seq dataset [36]. The analysis confirmed the results obtained with H3K27ac (Fig 3G). Rhythmic eRNA expression is only observed at CLOCK:BMAL1 enhancers targeting rhythmically transcribed genes, and eRNA expression at enhancers targeting non-expressed genes is dramatically decreased to levels close to background (Fig 3G). Importantly, these differences in eRNA expression between the four CLOCK:BMAL1 transcriptional output categories are further corroborated by similar variations in RNA Polymerase II (Pol II) ChIP-Seq signal at CLOCK:BMAL1 enhancers (S8C Fig). Altogether, our analysis therefore demonstrates that, contrary to what has been typically found for core clock genes, CLOCK:BMAL1 DNA binding is not sufficient to promote the activation of its enhancers. Instead, our results suggest that CLOCK:BMAL1 rhythmically opens the chromatin to facilitate the binding of other transcription factors at its enhancers, and that the nature of these transcription factors (e.g., activators, repressors) significantly contributes to CLOCK:BMAL1 transcriptional output.

### Differential recruitment of transcription factors at CLOCK:BMAL1 enhancers

To test our hypothesis that transcription factors bind at CLOCK:BMAL1 enhancers to contribute to their transcriptional activity and thereby impact on CLOCK:BMAL1-mediated transcription, we assessed if transcription factors were differentially recruited at CLOCK:BMAL1 DNA binding sites within each transcriptional output group. To this end, we performed a DNA binding motif analysis using HOMER Software Suite that we further validated using mouse liver transcription factor ChIP-Seq datasets.

**Fig 4:**
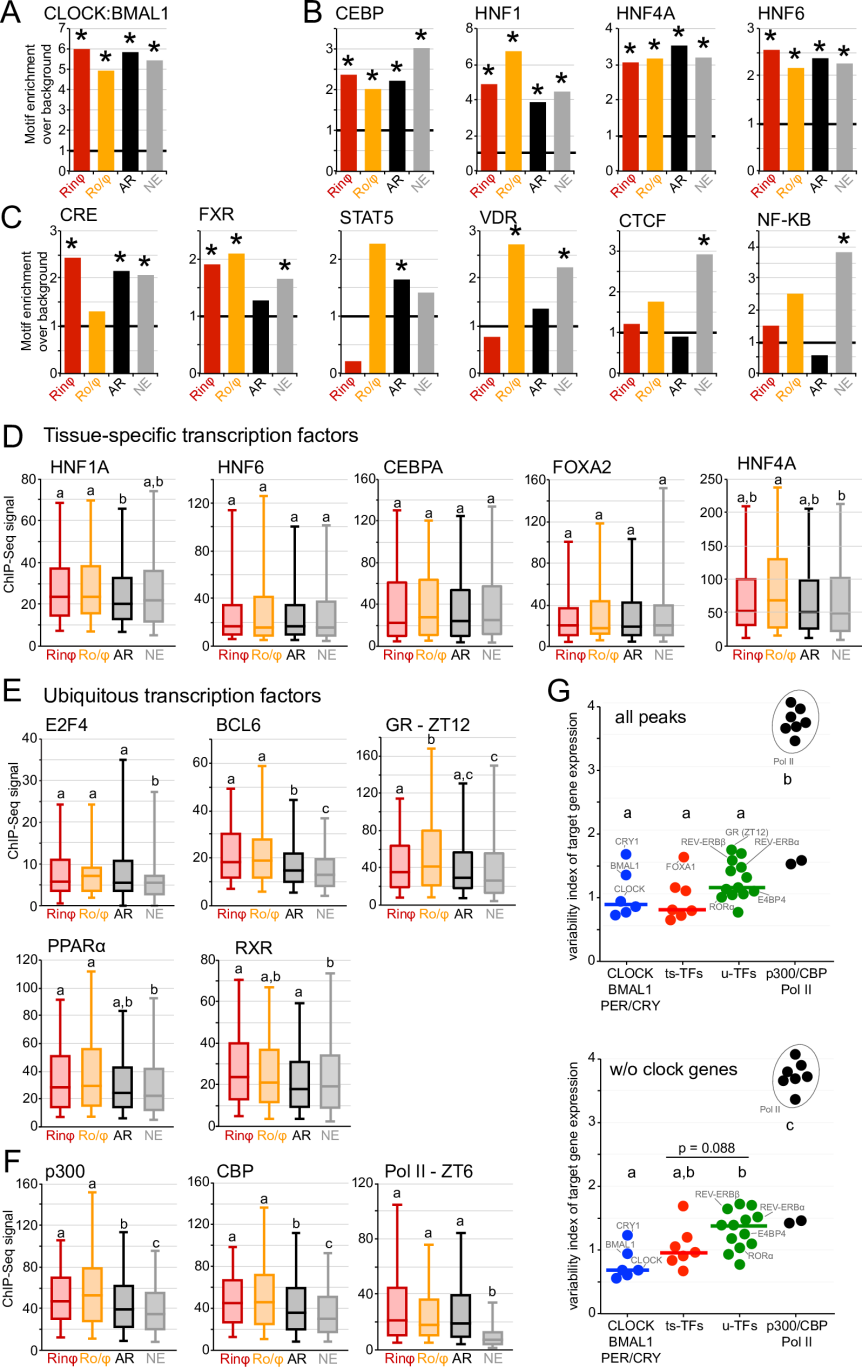
Tissue-specific and ubiquitous transcription factors are differentially recruited at CLOCK:BMAL1 enhancers. A-C. Enrichment for the DNA binding motif of CLOCK:BMAL1 (A), tissue-specific transcription factors (B) and ubiquitous transcription factors (C) at CLOCK:BMAL1 DNA binding sites for each of the four CLOCK:BMAL1 transcriptional output categories. Enrichment was calculated using HOMER and is reported as the ratio between the calculated enrichment over the calculated background. * q < 0.05 (Benjamini-Hochberg procedure). D-F. ChIP-Seq signal of tissue-specific transcription factors (D), ubiquitous transcription factors (E), and transcriptional co-activators / RNA Polymerase II at ZT6 (F) at CLOCK:BMAL1 DNA binding sites (peak center ± 250bp) for each of the transcriptional output categories. Groups with different letters are significantly different (Kruskal-Wallis test; p < 0.05). G. Transcription factor DNA binding variability index at CLOCK:BMAL1 DNA binding sites. The TF DNA binding variability index reflects differential TF DNA binding by calculating the variance of TF ChIP-Seq signal between the four CLOCK:BMAL1 transcriptional output groups (see methods for details). The variability index is displayed as a dot for each TF: CLOCK, BMAL1, PER1, PER2, CRY1, CRY2 (blue), seven ts-TFs (CEBPA, CEBPB, FOXA1, FOXA2, HNF1, HNF4A, and HNF6; red), thirteen u-TFs (REV-ERBα, REV-ERVβ, RORα, E4BP4, RXR, LXR, PPARα, GR-ZT12, E2F4, STAT5, BCL6, ERα, and GABPA; green), as well as for p300, CBP, and Pol II at seven time points (ZT02 to ZT26) (black). The horizontal lines represent the variability index median for the first 3 groups of TF. ChIP-Seq datasets used in this analysis are described in the method section. The variability index was calculated using all CLOCK:BMAL1 peaks analyzed in Fig 1 (left), or CLOCK:BMAL1 peaks that do not target a clock gene (removal of TF ChIP-Seq signal at peaks targeting *Per1*, *Per2*, *Cry2*, *Dbp*, *Rev-erbα*, and *Rev-erbβ*, *Tef*, *Hlf*, *Gm129*, and *Rorγ*; right).

As expected, the motif analysis revealed that CLOCK:BMAL1 DNA binding motif (e-box of the sequence CACGTG) is highly enriched at CLOCK:BMAL1 enhancers for all transcriptional output categories (Fig 4A). Surprisingly however, we found that motifs for liver-specific transcription factors (e.g., *Cebp*, *Hnf1*, *Hnf4* and *Hnf6*) were also enriched for all four transcriptional output categories, and thus even at CLOCK:BMAL1 enhancers targeting non-expressed genes (Fig 4B, S9 Fig, and S2 Table). On the contrary, motifs for ubiquitous transcription factors (u-TFs; broadly expressed transcription factors with a transcriptional activity regulated by external factors) were almost always enriched for specific CLOCK:BMAL1 transcriptional output groups (Fig 4C, S9 Fig, and S2 Table). For example, CRE motif was enriched at all CLOCK:BMAL1 enhancers except those targeting out-of-phase transcriptional cyclers, and FXR motif was enriched at all CLOCK:BMAL1 enhancers except those targeting out-of-phase transcriptional cyclers. Noticeably, the motifs for NF-κB [which binds to DNA and becomes transcriptionally active upon infection and inflammation; 41, 42], and CTCF [which establishes discrete functional chromatin domains by promoting DNA looping; 43, 44, 45] were enriched at enhancers targeting non-expressed genes.

To assess the relevance of this difference of motif enrichments between tissue-specific (ts-TFs) and u-TFs, we determined the DNA binding pattern of several transcription factors at CLOCK:BMAL1 enhancers in the mouse liver using publicly available transcription factors ChIP-Seq datasets [46–51]. This *in vivo* analysis largely confirmed the computational motif analysis: most liver-specific TFs were found to bind at CLOCK:BMAL1 DNA binding sites independently of the transcriptional output, whereas u-TFs were more specifically enriched in specific CLOCK:BMAL1 transcriptional output groups (Fig 4D, E, S10 Fig, and S3 Table). For example, *Hnf4a* and *Hnf1* are the only liver-specific TF to exhibit a differential binding between CLOCK:BMAL1 transcriptional output groups of the six TFs tested (*Foxa1*, *Foxa2*, *Hnf1*, *Hnf4A*, *Hnf6* and *Cepba*). Conversely, all twelve u-TFs investigated exhibit DNA binding differences at CLOCK:BMAL1 enhancers between categories of transcriptional output (Fig 4D and 4E, S10 Fig). Although each u-TF bound to different subsets of CLOCK:BMAL1 enhancers, u-TF recruitment was generally higher in rhythmically expressed target genes and lower in non-expressed target genes compared to the arrhythmic CLOCK:BMAL1 target group. (Fig 4D and 4E, S10 Fig, and S3 Table). To further characterize the differences in TF DNA binding between CLOCK:BMAL1, u-TFs and ts-TFs, we computed a TF DNA binding variability index by calculating the standard deviation of the ChIP-Seq signal between the 4 CLOCK:BMAL1 transcriptional output groups (see methods for details). We found that the DNA binding variability at CLOCK:BMAL1 peaks is comparable between CLOCK:BMAL1 and ts-TFs, whereas there is significantly more variability for u-TFs than for CLOCK and BMAL1 when peaks targeting core clock genes are removed from the analysis (Fig 4G). While there are variability index differences among ts-TFs and u-TFs, this analysis further supports our finding that u-TF recruitment at CLOCK:BMAL1 peaks is globally more variable than for ts-TF (Fig 4G).

### CLOCK:BMAL1 likely cooperates with other transcription factors to regulate the transcription of its direct target genes

**Fig 5:**
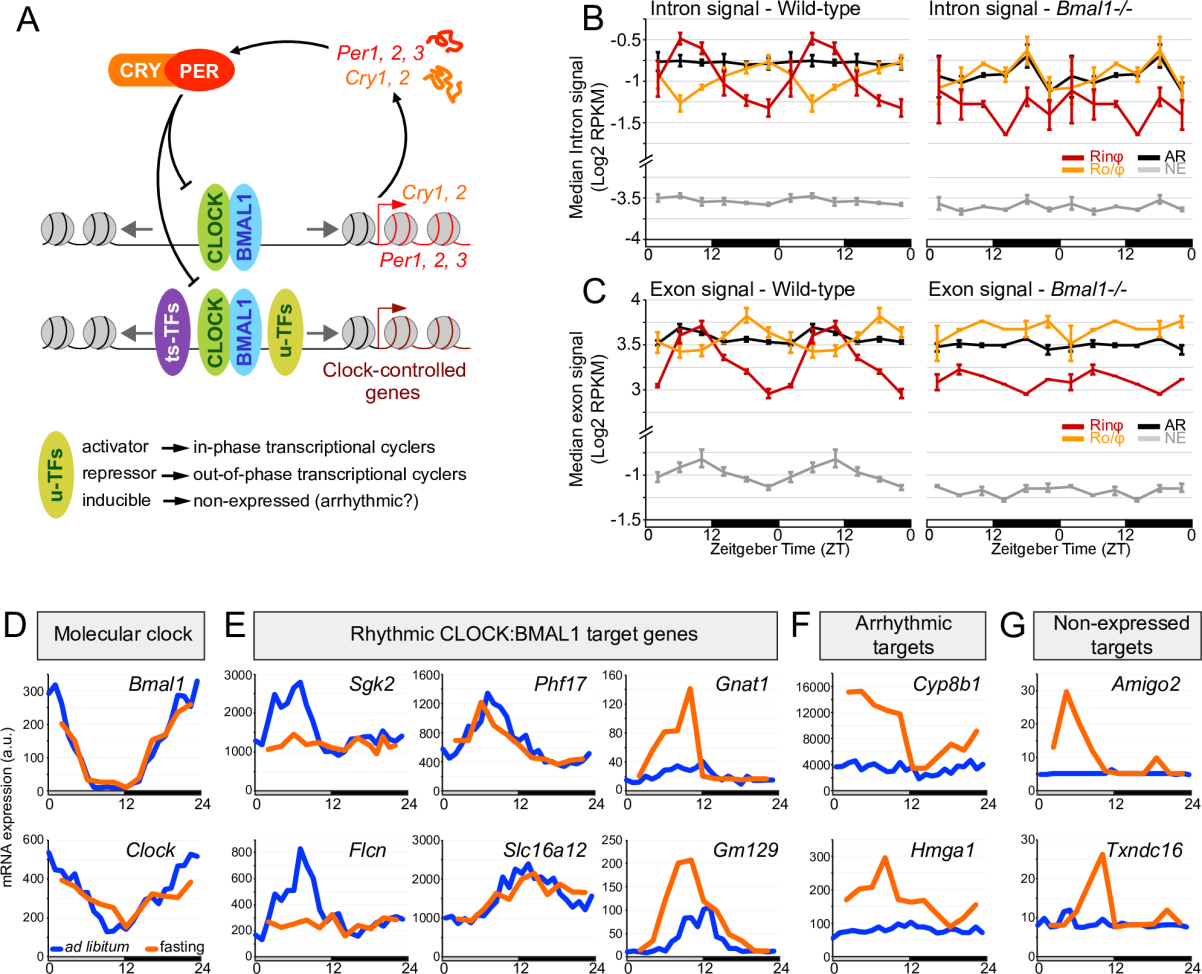
CLOCK:BMAL1 regulation of clock-controlled gene expression likely relies on the cooperation of CLOCK:BMAL1 with other transcription factors. A. Proposed model incorporating tissue-specific (ts-TFs) and ubiquitous (u-TFs) transcription factors into CLOCK:BMAL1 regulation of clock-controlled gene transcription. See text for details. B, C. Intron (B) and exon (C) signals of direct CLOCK:BMAL1 target genes classified based on their transcriptional output (Rinφ: in-phase transcriptional cyclers; Ro/φ: out-of-phase transcriptional cyclers; AR: arrhythmically transcribed target genes; NE: non-expressed target genes), in wild-type (left) and *Bmal1-/-* (right) mouse liver. Values correspond to the median RPKM for each transcriptional output group, and are displayed as the average ± s.e.m. of four (wild-type) or two (*Bmal1-/-*) independent samples for each time point. Data were retrieved from public RNA-Seq datasets [53], and are double-plotted for better visualization. D-G. Rhythm of mRNA expression in the liver of mice fed *ad libitum* (blue) or fasted for at least 22 hours (orange). Data were retrieved from a public dataset [56]. Mouse liver mRNA expression is displayed for *Clock* and *Bmal1* (D), as well as CLOCK:BMAL1 target genes that are rhythmically expressed (E), arrhythmically expressed (F), or not expressed (G) in the liver of mice fed *ad libitum*.

Altogether, our data indicate that the mechanisms by which CLOCK:BMAL1 regulates transcription of clock-controlled genes differ from the well-characterized CLOCK:BMAL1-mediated regulation of core clock gene expression. Specifically, our data show that although CLOCK:BMAL1 mediates rhythmic nucleosome removal at its enhancers, it is not sufficient to generate an active enhancer or drive rhythmic transcription. We thus propose a model whereby CLOCK:BMAL1 regulates transcription of clock-controlled genes by rhythmically opening chromatin to facilitate the binding of other transcription factors at its enhancers (Fig 5A). This possibility is supported by results showing that nucleosome signal is rhythmic at the DNA binding sites of several TFs when those sites are located close to a CLOCK:BMAL1 peak, and not rhythmic when CLOCK:BMAL1 binding is absent (S11 Fig). Consequently, the transcriptional activities of these transcription factors would dictate the transcriptional outcome of clock-controlled genes rather than CLOCK:BMAL1 (Fig 5A). For example, binding of positive transcription factors along with CLOCK:BMAL1 would activate enhancers and lead to transcription activation during the day, whereas binding of transcriptional repressors (e.g., REV-ERBα/β) would inhibit CLOCK:BMAL1 enhancer activity and thereby contribute to rhythmic transcription peaking during the night, in anti-phase with CLOCK:BMAL1 DNA binding (Fig 5A). If no transcription factors are recruited (e.g., inducible transcription factors), CLOCK:BMAL1 enhancers remain silent and target genes are not expressed or are arrhythmically expressed (Fig 5A). Arrhythmically expressed genes at CLOCK:BMAL1 enhancers may also have positive transcription factors bound at all times overriding the absence of CLOCK:BMAL1 DNA binding at night (see result section about rhythmic nucleosome signal and Fig 3E). Our results also suggest that u-TFs regulate CLOCK:BMAL1 transcriptional output more prevalently than ts-TFs. It may be that ts-TFs facilitate the binding of CLOCK:BMAL1 at tissue-specific enhancers rather than contributing to CLOCK:BMAL1 transcriptional output (see discussion). To validate this model experimentally, we investigated how i) *Bmal1* knockout, and ii) changes in environmental conditions (that alter u-TFs transcriptional activities) affect CLOCK:BMAL1 transcriptional output.

If the activity of u-TFs contributes to CLOCK:BMAL1 regulation of clock-controlled gene transcription, then, a knockout of *Bmal1* (which eliminates CLOCK:BMAL1-mediated transcription [52]) should differentially affect the expression of CLOCK:BMAL1 target genes. Specifically, target gene expression levels in *Bmal1-/-* mouse should be arrhythmic and low for the in-phase transcriptional cyclers (no recruitment of positive transcription factors at CLOCK:BMAL1 enhancers), while they should be arrhythmic and high for the out-phase transcription cyclers (no recruitment of repressors at CLOCK:BMAL1 enhancers). These effects should also be more obvious during the light phase when CLOCK:BMAL1 binds to DNA. In addition, *Bmal1* knockout should have a reduced effect on arrhythmically and non-expressed target genes. These predictions were confirmed by analyzing a public dataset that characterized the genome-wide effect of *Bmal1* knockout in the mouse liver using RNA-Seq of rRNA-depleted total RNA (Fig 5B and 5C) [53]. For both intronic and exonic RNA-Seq signal, the expression of Rinφ genes in *Bmal1-/-* mouse liver is at the trough level of wild-type mice, and at peak levels in Ro/φ genes. Moreover, *Bmal1* knockout does not significantly affect the expression levels of arrhythmic and non-expressed CLOCK:BMAL1 target genes (Fig 5B and 5C).

Our model also predicts that the transcriptional output of CLOCK:BMAL1 target genes can be altered by environmental changes that affect u-TF DNA binding capacity. External signals that activate or repress the binding of u-TFs are predicted to impact CLOCK:BMAL1 cooperation with other transcription factors, and thereby change the transcriptional output of CLOCK:BMAL1 target genes. For example, signals that enable the recruitment of positive transcription factors at CLOCK:BMAL1 enhancers could increase the amplitude of rhythmic transcription and/or initiate the rhythmic expression of target genes that are arrhythmic under control conditions. Conversely, signals that inhibit the binding of transcription factors that normally cooperate with CLOCK:BMAL1 could blunt the rhythmic expression of some CLOCK:BMAL1 target genes. To test this hypothesis, we analyzed how fasting, which is known to affect the transcriptional activity of many u-TFs [54, 55], alters CLOCK:BMAL1 target gene expression in the mouse liver using a public dataset [56]. Strikingly, while the expression of *Clock*, *Bmal1* and several direct rhythmic target genes (e.g., *Phf17*, *Slc16a2*) are unaffected by fasting, some other targets exhibit a significantly altered gene expression profile (Fig 5D–5G, S12 Fig for additional examples). For example, some rhythmic target genes become arrhythmically expressed under fasting (e.g., *Sgk2*, *Flcn*) while other targets exhibit an increased amplitude of expression (e.g., *Gnat1*, *Gm129*) (Fig 5E). Remarkably, some direct CLOCK:BMAL1 target genes that are arrhythmically or not expressed under *ad libitum* condition become rhythmically expressed under fasting condition (Fig 5F and 5G). Because not all CLOCK:BMAL1 target genes are equally affected by fasting, these results cannot simply be explained by a global change in CLOCK:BMAL1 transcriptional activity under fasting condition. One possibility is that fasting enhances or represses the transcriptional capabilities of several u-TFs that cooperate with CLOCK:BMAL1, thereby altering the transcriptional output of many direct CLOCK:BMAL1 target genes. Similar results were found by analyzing a public dataset investigating the effect of high-fat diet on rhythmic gene expression in the mouse liver (S13 Fig) [57].

## Discussion

Based on the mechanisms by which CLOCK:BMAL1 regulates the expression of several core clock genes, it is commonly assumed that the rhythmic binding of CLOCK:BMAL1 to DNA is necessary and sufficient to drive the rhythmic transcription of its target genes. However, the recent characterization of CLOCK and BMAL1 cistromes in the mouse liver revealed that CLOCK:BMAL1 target gene transcription is highly heterogeneous, thereby suggesting that CLOCK:BMAL1 regulation of clock-controlled gene expression relies on more complex mechanisms than those underlying core clock gene rhythmic transcription [3, 28–30]. We report here that CLOCK:BMAL1 heterogeneous transcriptional output does not stem from differences in the DNA binding profiles of CLOCK and BMAL1, or the PER/CRY circadian repressive complex. Instead, we found that while CLOCK:BMAL1 rhythmically promotes chromatin decondensation at its enhancers, it is not sufficient to promote transcription activation. Based on these data and the characterization of transcription factor DNA binding profiles at CLOCK:BMAL1 enhancers, we propose that CLOCK:BMAL1 regulates the expression of clock-controlled genes by generating a permissive chromatin landscape that facilitates the binding of other transcription factors at its enhancers rather than directly promoting rhythmic transcription. Interestingly, analysis of a random set of genes not directly targeted by CLOCK:BMAL1 but exhibiting similar profiles of expression of the four CLOCK:BMAL1 transcriptional output categories suggests that this mechanism is largely specific to CLOCK:BMAL1 (S14 Fig).

The current models describing the regulation of rhythmic gene expression by circadian clocks in other eukaryotes are also based on how core clock components regulate their own transcription via transcriptional feedback loops. For example, the mechanisms underlying transcriptional regulation by CLOCK:BMAL1 orthologs in *Neurospora* (WCC for White Collar Complex) and *Drosophila* (CLK:CYC heterodimer) are based largely on how they regulate the expression of the core clock genes *frequency* (in *Neurospora*), and *period* and *timeless* (in *Drosophila*) [2, 58–60]. Given that the circadian clock mechanisms are highly conserved in eukaryotes, it is likely that both WCC and CLK:CYC also regulate their target gene expression by remodeling the chromatin and facilitating the binding of other transcription factors. Consistent with this hypothesis, WCC and CLK:CYC transcriptional outputs are also heterogeneous [61, 62], and both recruit chromatin remodelers to promote nucleosome eviction at their binding sites [63–67].

The recent characterization of many transcription factor cistromes revealed that the number of transcription factor DNA binding sites often exceeds the number of anticipated target genes, suggesting that many of these DNA binding sites are non-functional [68, 69]. Although many CLOCK:BMAL1 DNA binding sites could be considered as non-functional because they target arrhythmically or not expressed genes, the observation that CLOCK:BMAL1 rhythmically promotes nucleosome eviction at enhancers targeting both arrhythmically expressed (albeit with a decreased amplitude) and non-expressed genes instead indicates that CLOCK:BMAL1 rhythmic DNA binding is not “silent”. More specifically, our data suggest that the majority of CLOCK:BMAL1 DNA binding events are functional, in that they rhythmically shape the chromatin landscape, and that transcription activation requires additional downstream events to be initiated (e.g., recruitment of other transcription factors). This hypothesis is further supported by our finding that CLOCK:BMAL1 does not directly generate a transcriptionally active enhancer. Indeed, both H3K27ac ChIP-Seq signal and eRNA transcription are minimal at CLOCK:BMAL1 enhancers targeting non-expressed genes, and are delayed at CLOCK:BMAL1 enhancers targeting out-of-phase transcriptional cyclers (Fig 3F and 3G). The observation that H3K27ac ChIP-Seq signal at CLOCK:BMAL1 enhancers correlates with CLOCK:BMAL1 transcription output rather than CLOCK:BMAL1 DNA binding phase/intensity seems inconsistent with the well-described interactions between core clock proteins and histone modifiers [3, 14–19, 26], and thus raises the question on whether or not CLOCK:BMAL1 DNA binding occurs with enzymatically activate histone modifiers. Interestingly, instances of enhancers bound by p300/CBP but lacking H3K27ac (and transcriptional activity) have been described at enhancers targeting developmental genes in human ES cells [70, 71]. Those enhancers, which are termed poised enhancers, share most of the properties of active enhancers, including similar levels of nucleosome depletion, p300, and chromatin remodelers binding. However, these poised enhancers are unable to drive gene expression in ES cells until they acquire H3K27ac signal during differentiation [71]. Here we found that the binding of CBP and p300 at non-expressed target genes is above background levels, and that the differences in CBP and p300 DNA binding between non-expressed and expressed target genes are smaller than those observed for H3K27ac and Pol II ChIP-Seq signal (Figs 3Fand 4F, S10 Fig). It is thus tempting to speculate that the concept of poised enhancers extends to the circadian field, with CLOCK:BMAL1 rhythmically priming the chromatin landscape of “circadian poised enhancers”. While those circadian poised enhancers would share properties of active enhancers (similar CLOCK:BMAL1 DNA binding, nucleosome eviction rhythm, *etc*.), they would be transcriptionally inactive and require the binding of other transcription-associated factors needed to trigger H3K27ac and rhythmic transcription.

Investigation of the transcription factors that are recruited at CLOCK:BMAL1 enhancers revealed a surprising difference between u-TFs and ts-TFs. In particular, ts-TFs are recruited at similar levels between expressed and non-expressed CLOCK:BMAL1 target genes, suggesting that they do not significantly contribute to the heterogeneity of CLOCK:BMAL1 transcriptional output. Because ts-TFs are known to establish tissue-specific enhancers and enable the binding of u-TFs in a tissue-specific manner [72–75], it is likely that ts-TFs contribute primarily to the binding of CLOCK:BMAL1 at tissue-specific enhancers and thus enable the generation of a tissue-specific circadian transcriptional program [8–10, 76]. Contrary to ts-TFs, u-TFs appear to bind at CLOCK:BMAL1 enhancers targeting specific transcriptional output categories, suggesting that their nature (i.e., activator or repressor, constitutively active or inducible), as well as mode of cooperation with CLOCK:BMAL1, likely contributes to the heterogeneity of CLOCK:BMAL1 target gene transcription. For example, the transcriptional repressors REV-ERBα and REV-ERBβ are enriched at CLOCK:BMAL1 enhancers targeting out-of-phase transcriptional cyclers, agreeing with the recently proposed model of facilitated repression whereby CLOCK:BMAL1 remodels its enhancer chromatin to facilitate the recruitment of REV-ERBs and delay the transcriptional output of some of its target genes [37]. Since rhythmically expressed genes tend to exhibit higher u-TF ChIP-Seq signal than arrhythmic and non-expressed genes (S10 Fig), and given the low expression of in-phase transcriptional cyclers in *Bmal1-/-* mice, we propose that a major function of CLOCK:BMAL1 is to facilitate the recruitment of both positive and negative transcription factors to drive the rhythmic transcription of clock-controlled genes (i.e., not just to facilitate the binding of the circadian repressors REV-ERBα/β). Although the mechanisms underlying of this cooperation between CLOCK:BMAL1 and other transcription factors are still unknown, nucleosome-mediated cooperation between transcription factors is not unprecedented [77–82], and several papers have shown that two non-interacting TFs can synergistically bind to DNA through a mechanism whereby the first TF leads to partial unwrapping of nucleosomal DNA, thus making the site of the second TF more accessible and thereby increasing DNA binding.

This cooperation between CLOCK:BMAL1 and other TFs may explain why a large fraction of CLOCK:BMAL1 target genes are not expressed: u-TF recruitment is not sufficient to activate CLOCK:BMAL1 enhancers and promote transcription. In support of this idea, CLOCK:BMAL1 enhancers targeting non-expressed target genes are enriched for the NF-κB transcription factor motif, which is known to mediate transcriptional response to immune and inflammatory responses [41]. Because the genome-wide characterization of circadian clock mechanisms has mostly been carried out in healthy mice raised in standard laboratory conditions, NF-κB is likely inactive, sequestered in the cytosol and its target genes are not expressed. CLOCK:BMAL1 may thus prime NF-κB DNA binding upon inflammation or immune response, thereby triggering a rhythmic response to acute infection. Interestingly, such a mechanism may explain, at least in part, why the immune host response oscillates based on the time-of-day bacterial infection [83–86]. We also found that CLOCK:BMAL1 enhancers at non-expressed target genes are enriched for the transcription factor CTCF (CCCTC-binding factor; Fig 4A). CTCF is known to promote long-range interactions between two or more genomic sequences, and thus bring sequences that are far apart in the linear genome into close proximity [44]. This may suggest that some CLOCK:BMAL1 DNA binding sites situated in non-expressed gene loci actually target other clock-controlled genes located hundreds of kilobases apart through long-range interactions, as recently described for one CLOCK:BMAL1 DNA binding site in the mouse liver [87]. Although it is impossible to assess the prevalence of CLOCK:BMAL1 binding sites mediating long-range chromatin interactions without the appropriate experiments, we found a few examples suggesting that this is a likely possibility (S15 Fig).

Transcription regulation in higher eukaryotes relies on the activity of multiple enhancers [88, 89]. It is thus likely that CLOCK:BMAL1 target gene expression results from a complex integration between CLOCK:BMAL1 enhancers and other enhancers. Our results indicate that enhancers targeting the same gene typically share the same transcriptional activity profiles (H3K27ac signal, eRNA levels, and Pol II ChIP-Seq signal; S8 Fig). Based on these observations, we cannot exclude that other enhancers targeting arrhythmically expressed CLOCK:BMAL1 target genes outcompete CLOCK:BMAL1 enhancers, to produce constitutive expression. Further experiments aimed at revealing hierarchical influences of enhancers on the regulation of gene expression at the genome-wide level will be required to directly test this hypothesis.

It was recently proposed that altering the environmental conditions can reprogram circadian transcriptional programs (e.g., high-fat diet and antibiotics treatment in the liver, LPS treatment in the lung [57, 90–92]). Our model that CLOCK:BMAL1 regulates the expression of clock controlled genes by facilitating the binding of other TFs represents a mechanistic framework for explaining how environmental signals can mediate this transcriptional reprogramming. Indeed, activation of new signaling pathways by environmental changes is likely to modulate multiple transcriptional programs, thereby altering how CLOCK:BMAL1 cooperate with those programs to drive rhythmic gene expression. Importantly, this mechanism may also explain, at least in part, why the number and nature of rhythmically expressed genes vary between datasets and laboratories [93–95]. Indeed, differences in diet, light environment and housing may all lead to changes in u-TF transcriptional activity, which may in turn affect clock-controlled gene expression.

In conclusion, our data indicate that the mechanisms by which CLOCK:BMAL1 regulates the transcription of core clock genes do not apply to clock-controlled genes, and suggest that the primary function of CLOCK:BMAL1 is to regulate the chromatin landscape at its enhancers to facilitate the binding of other transcription factors. Our results therefore highlight the emerging role of other transcription factors in regulating the ~15% of genes that are rhythmically expressed in a given mammalian tissue, and suggests that clock-controlled gene expression relies more on the interplay between the circadian clock and other signaling pathways. Given that the majority of CLOCK:BMAL1 target genes are either arrhythmically or not expressed under standard conditions, our data also suggest that these non-oscillating genes may become rhythmically expressed under other environmental and/or pathological conditions, and thus expand the total number of genes under circadian control to more than 50% in mammals [10]. Finally, because the clockwork mechanisms are highly conserved between eukaryotes (e.g., heterogeneous transcriptional output, poor reproducibility between datasets characterizing circadian gene expression, regulation of chromatin landscape by core clock components), it is likely that the mechanisms we uncovered largely apply to all eukaryotic circadian clocks.

## Materials and methods

### Sequencing datasets and alignment to the mouse genome

Unless notified below, publically available datasets used in this paper were downloaded from the NCBI or EBI websites in either sra or fastq formats (see Table 1 for accession numbers). Files in sra format were converted to fastq files using the sratoolkit (version 2.3.5–2). Fastq files were mapped to the mouse genome (version mm10) using bowtie2 [96] or tophat2 [97]. For all datasets, we only considered reads that mapped uniquely to the mouse genome (i.e., one unique genomic location). Datasets were further filtered to remove duplicated reads using samtools (rmdup function) or a custom-made script. Additional information is provided for each dataset as Supplementary Materials and Methods.

**Table 1:** Public datasets used in this study.

Reference	SRA/GEO dataset	Data	Remapped
Koike et al., 2012 [3]	GSE39977	ChIP-Seq: BMAL1, CLOCK, PER1, PER2, CRY1,CRY2, H3K27ac	Yes^1^
Menet et al., 2012 [28]	GSE36916	Nascent-Seq RNA-Seq	Yes^2^
Rey et al., 2011 [29]	GSE26602	ChIP-Seq: BMAL1	Yes
Cho et al., 2012 [35]	GSE34020	ChIP-Seq: Reverba, Reverbb	Yes
Menet et al., 2014 [20]	GSE47145	MNase-Seq	Yes^3^
Le Martelot et al., 2012 [22]	GSE35790	ChIP-Seq: RNA polymerase II (Pol II)	Yes
Vollmers et al., 2012 [38]	SRA025656	ChIP-Seq: H3K9ac, H3K27ac	Yes
Fang et al., 2014 [36]	GSE59486	ChIP-Seq: E4BP4, Roralpha GRO-Seq	Yes^4^
Ling et al., 2010 [98]	GSE21777	DNase-Seq	No^5^
Faure et al., 2012 [46]	https://www.ebi.ac.uk/arrayexpress/experiments/E-MTAB-941/	ChIP-Seq: GABPA, HNF1, HNF4A, E2F4, CEBPA, HNF6, p300, CBP, CTCF	Yes
Lim et al., 2015 [47]	GSE59752	ChIP-Seq: CEBPB, GR	Yes
Boergesen et al., 2012 [48]	GSE35262	ChIP-Seq: LXR, PPARa, RXR	Yes
Gordon et al., 2014 [50]	GSE52351	ChIP-Seq: ERalpha	Yes
MacIsaac et al., 2010 [49]	GSE17067	ChIP-Seq: FOXA1, FOXA2	Yes
Zhang et al., 2012 [51]	GSE31578	ChIP-Seq: BCL6, STAT5	Yes
Atger et al., 2015 [53]	GSE73554	RNA-Seq: Wild-type and Bmal1-/- mouse liver	No^6^
Eckel-Mahan et al., 2015 [57]	GSE52333	Mouse liver RNA expression (microarray data) from wild-type mice fed normal chow or high fat diet	No^7^
Vollmers et al., 2009 [56]	GSE13093, GSE13064	Mouse liver RNA expression (microarray data) from wild-type mice fed *ad libitum* or subjected to fasting for 24 hours.	Yes

^1^ Datasets from Koike et al., 2012 were downloaded as sra files and remapped to the mouse genome (mm10 version) to analyze ChIP-Seq signal at various enhancers. However, we used the CLOCK and BMAL1 peak coordinates provided in the S2 Table of the original paper to generate our list of high confidence CLOCK:BMAL1 DNA binding sites (see details below), and also determine the phase and intensity of CLOCK and BMAL1 DNA binding.

^2^ Nascent-Seq and RNA-Seq dataset were remapped to the mouse genome version mm10 (e.g., to generate the S14 Fig). However, Nascent-Seq and RNA-Seq signals were retrieved from the original paper (Fig 2—source data 1 and Fig 3—source data 2) and used to generate the data presented in Fig 1.

^3^ While fastq files were remapped to the mouse genome version mm10, further analysis was performed as described in the original paper [20]. For example, the 50nt reads were extended to 147nt to match the length of a mononucleosome.

^4^ Analysis of the GRO-Seq dataset has been performed similarly to the original paper and reads were also extended to 150bp as in Fang et al., 2014 [36].

^5^ No reanalysis of the DNase-Seq was performed. The file GSE21777_M-CM_peaks.txt, which contain the list of DNaseI hypersensitive sites (DHS) in the mouse liver, was downloaded from the ncbi website (GSE21777) and the DHS peak coordinates (mouse genome version mm9) were converted to mouse genome version mm10 using a liftOver tool downloaded from the UCSC genome browser website (conversion resulted in a list of 104,556 DHS peaks).

^6^ The processed files with intron and exon RNA-Seq signal (fpkm) from wild-type and *Bmal1-/-* mouse liver were downloaded from the ncbi website and directly used to generate the Fig 5B and 5C (files GSE73554_KO_RF_Intron_Exon_RFP.txt and GSE73554_WT_RF_Intron_Exon_RFP.txt).

^7^ Processed microarray data were downloaded from the ncbi website and used to generate the Fig 5D–5G. The original statistical analysis performed by the authors and provided as supplementary S2 Table from the journal Cell website was used in our study to assess rhythmic gene expression.

### Identification of CLOCK:BMAL1 DNA binding sites

Genomic locations of CLOCK and BMAL1 DNA binding sites in the mouse liver provided in the original paper (supplementary S2 Table) [3] were used to generate our list of high confidence CLOCK:BMAL1 DNA binding sites. Genomic locations were converted to the mm10 version of the mouse genome using UCSC genome browser liftOver tools, and processed as indicated in S1 Fig to generate our list of high confidence CLOCK:BMAL1 DNA binding sites. Overlap between CLOCK and BMAL1 ChIP-Seq peaks was determined using bedtools (intersectBed) and coordinates from BMAL1 ChIP-Seq datatsets were further kept to generate a list of 3217 CLOCK:BMAL1 peaks. We also used the original data provided by the authors in their S2 Table to assign CLOCK:BMAL1 peaks to their putative target genes (original analysis performed using HOMER tools). In particular, we defined a CLOCK:BMAL1 target gene as a gene with at least one CLOCK:BMAL1 peak located between -10kb of the transcription start site and +1kb from the transcription termination site. Using this criteria, 2458 CLOCK:BMAL1 peaks were assigned to a target gene, and the remaining 759 peaks were assigned as an intergenic CLOCK:BMAL1 DNA binding site.

The 2458 CLOCK:BMAL1 peaks assigned to a target gene were then parsed based on the transcription profile of their target genes using the Nascent-Seq datasets from Menet et al., 2012 [28]. We directly used the original Nascent-Seq expression values and the assessment of their rhythmic expression from the original paper without performing new analysis. Details on how genes were determined to be rhythmically transcribed are provided in Supplementary Materials and Methods. Using these data, CLOCK:BMAL1 peaks were parsed into 4 different categories of transcriptional output (see also S1 Fig):

(i) Rhythmic transcription in phase with CLOCK:BMAL1 binding (peak of transcription between ZT02 and ZT12), 205 CLOCK:BMAL1 peaks,
(ii) Rhythmic transcription out-of-phase with CLOCK:BMAL1 binding (peak of transcription between ZT12 and ZT02), 124 CLOCK:BMAL1 peaks,
(iii) Arrhythmic transcription, 654 CLOCK:BMAL1 peaks (criteria used to defined arrhythmic transcription are detailed in Supplementary Materials and Methods). Note that to decrease the number of false positive in the list of arrhythmically expressed genes, we removed genes that exhibit arrhythmic nascent RNA expression, but exhibit rhythmic mRNA expression (using the RNA-Seq dataset from Menet et al., 2012 [28],
(iv) Not transcribed (average reads/bp between the 12 sample < 1), 291 CLOCK:BMAL1 peaks.
The remaining peaks, which were not analyzed in this study, were categorized as:
(v) Post-transcriptional cyclers, 262 CLOCK:BMAL1 peaks. These peaks target genes with an arrhythmic Nascent-Seq signal, but rhythmic RNA-Seq signal (based on the reads/bp values published in Menet et al., 2012 datasets [28]).
(vi) Low expression levels, 588 CLOCK:BMAL1 peaks. These CLOCK:BMAL1 peaks target genes with Nascent-Seq and/or RNA-Seq signals that are below threshold for the analysis of rhythmic expression, but above the threshold of 1 read/bp set to define the “not-expressed” CLOCK:BMAL1 target genes. These thresholds were defined in Menet et al., 2012 [28].
(vii) No signal value, 334 CLOCK:BMAL1 peaks. These peaks were assigned to a target gene (defined as described above with the HOMER software and localization criteria), but no information was found in the Nascent-Seq or RNA-Seq. Several of these peaks target genes encoding for a non-coding RNA, as well as genes with alternative gene symbol.

The list of the 3217 CLOCK:BMAL1 peaks parsed into the different transcriptional output categories is provided in S1 Table. The phase of rhythmic CLOCK:BMAL1 DNA binding, ChIP-Seq signal, and genomic location of CLOCK:BMAL1 DNA binding sites were retrieved from the Koike et al., 2012 original paper supplementary S2 Table [3] and processed to generate the analysis presented in Fig 1.

### Analysis of ChIP-Seq, MNase-Seq and GRO-Seq signal at CLOCK:BMAL1 peaks

ChIP-Seq, MNase-Seq and GRO-Seq signal was retrieved from bam files containing uniquely mapped reads (and duplicated reads removed) at CLOCK:BMAL1 enhancers using custom-made scripts [20]. Specifically, signal was retrieved at:

- CLOCK:BMAL1 peak center ± 250 bp for transcription factors, CBP, p300 and Pol II,
- CLOCK:BMAL1 peak center ± 1kb for histone modifications,
- CLOCK:BMAL1 peak center ± 500 bp for eRNA,
- CLOCK:BMAL1 peak center ± 75bp for nucleosome signal,

and normalized to the sequencing depth. Differences in the window size were calculated based on the width of the ChIP-Seq signal at CLOCK:BMAL1 DNA binding sites (e.g., H3K27ac ChIP-Seq signal is significantly wider than any transcription factor ChIP-Seq signal). Because we aimed at assessing the role of CLOCK:BMAL1 in removing a nucleosome at its DNA binding site, we chose a narrower window size of 150bp (see Fig 3A–3D). All analyses were performed at individual CLOCK:BMAL1 ChIP-Seq peaks, and this even for peaks targeting the same gene. Data presented in S4 Fig examined the role of multiple peaks targeting the same gene on BMAL1 and CLOCK ChIP-Seq signals. For all datasets, ChIP-Seq signal is displayed as the number of reads/bp per 100,000,000 reads.

### Analysis of ChIP-Seq and GRO-Seq signal at other enhancers targeting CLOCK:BMAL1 target genes

Enhancers lying into CLOCK:BMAL1 target gene loci (-10kb from the transcription start site to +1kb from the transcription termination site) were identified using a public mouse liver DNAse-Seq dataset (see above) [98] and bedtools (intersectBed function). Enhancers were then parsed based on the presence or not of a CLOCK:BMAL1 ChIP-Seq peak (3155 out of the 3217 CLOCK:BMAL1 ChIP-Seq peaks are located into a DNaseI hypersensitive site). Because a majority of the 104,556 DHS peaks only displayed low levels of ChIP-Seq (transcription factors, Pol II, H3K27ac) and GRO-Seq signals [as shown in the ENCODE project, 99, 100], we filtered the number of DHS lying into a CLOCK:BMAL1 target gene by only considering those being into the top 40,000 DHS list (based on DNase-Seq signal), obtaining the following number of DHS peaks:

(i) In-phase transcription cyclers: 1548 DHS peaks,
(ii) Out-of-phase transcription cyclers: 1189 DHS peaks,
(iii) Arrhythmically expressed target genes, 5830 DHS peaks,
(iv) Not expressed target genes, 991 DHS peaks.

H3K27ac and Pol II ChIP-Seq signals, as well as GRO-Seq (eRNA) signal, were retrieved at those DHS sites (as well as those overlapping with a CLOCK:BMAL1 peak) using the DHS peak coordinate and normalized to 100,000,000 reads. Signal was then normalized to the coordinate length (in bp) to obtain the signal displayed as reads/bp per 100,000,000 reads. The coordinates used were, for the same reason as above for CLOCK:BMAL1 DNA binding sites:

- H3K27ac ChIP-Seq: DHS genomic coordinate center ± 1 kb,
- Pol II ChIP-Seq: DHS “real” genomic coordinates,
- GRO-Seq (eRNA signal): DHS genomic coordinate center ± 500 bp.

Because our analysis revealed the existence of small but significant overall variations of H3K27ac and Pol II ChIP-Seq signal between time points (see S8 Fig), we further normalized the datasets by performing either a mean normalization (H3K27ac) or a ranking analysis (Pol II). For H3K27ac ChIP-Seq datasets [3, 38], averaged H3K27ac signal was calculated at the top 40,000 DHS peaks (the top 40,000 DHS peaks concentrate the majority of TFs DNA binding sites; peak center ± 1 kb; total of 104,556 total DHS peaks; dataset from Ling et al., 2010 [98]) for each time point. This averaged signal was then used to normalize the raw H3K27ac ChIP-Seq signal, by calculating for each time point the ratio between H3K27ac signal for each peak and this averaged signal (see S8 Fig). Pol II ChIP-Seq dataset [22] were normalized by performing a ranking normalization (method similar to a quantile normalization). To this end, Pol II ChIP-Seq signal was calculated at all 104,556 DHS peaks (peaks mapped in Ling et al., 2010 paper [98]), and sorted based on the ChIP-Seq values. The raw values for each DHS peak were then normalized using the sorted averaged ChIP-Seq signal at each of the 104,556 ranks for all time points.

### Motif analysis

Motif analysis was performed at CLOCK:BMAL1 enhancers (original peak coordinates) for each of the transcriptional categories using the findsMotifGenome.pl script from the HOMER suite. Parameters were as the following: -size given–len8. The resulting table was sorted by the q-value and a q-value less than 0.05 was considered significant. Percent enrichment (percent of target sequences with motifs / percent of background sequences with motif) was then calculated for motifs found to be significant in at least one of the CLOCK:BMAL1 transcriptional output category. Results of the motif analysis are provided as S2 Table.

### Determination of the TF DNA binding variability index

To determine the variance of each TF DNA binding (CLOCK, BMAL1, ts-TFs and u-TFs) between the four CLOCK:BMAL1 transcriptional output categories, we computed a TF DNA binding “variability index” based on the analysis performed in S10 Fig. The variability index was calculated by summing up the standard deviation of the ChIP-Seq signal between the 4 transcriptional output groups, which was calculated for each decile (0.1 to 0.9) and normalized to the averaged signal for each decile (the standard deviation is higher for upper deciles because ChIP-Seq signals are higher). This index reflects differential DNA binding strength between groups, as similar binding between the 4 groups results in small standard deviation values for each decile, and thus a small variability index. Conversely, differences in DNA binding signal between groups result in larger standard deviation values and thus a larger variability index.

### Generation of a list of control genes not targeted by CLOCK:BMAL1

To determine if the results described in this paper are specific to CLOCK:BMAL1, we also performed an analysis on genes not targeted by CLOCK:BMAL1, but exhibiting similar profiles of expression to the 4 CLOCK:BMAL1 transcriptional output categories (Rinφ, Ro/φ, AR and NE). To this end, 125 genes were randomly selected for each of the 4 groups, using criteria similar to those used to define CLOCK:BMAL1 transcriptional output (see above). Levels of expression for each group were not significantly different to those of CLOCK:BMAL1 target genes (Kruskal-Wallis test). Nucleosome signal, H3K27ac ChIP-Seq signal, Pol II ChIP-Seq signal, eRNA expression, tissue specific and ubiquitous transcription factor ChIP-Seq signal were all calculated as described above for CLOCK:BMAL1 target genes. Statistical analysis was also performed similarly to CLOCK:BMAL1 transcriptional output.

### Statistical analysis

Statistical analysis was done using JMP, Version *Pro 12*.*0*.*1*. SAS Institute Inc., Cary, NC, 1989–2007. Differences in sequencing signal, represented in the boxplot graphs, were analyzed for statistical enrichment using the nonparametric Kruskal-Wallis test. Rhythmic analysis of nucleosome signal and ChIP-Seq signal was performed using a Fourier analysis (Fig 3A–3D) (see Supplementary Materials and Methods for details). Differences in the amplitude of nucleosome signal rhythm (Fig 3E) were analyzed using a 2-way ANOVA. Differences in CLOCK:BMAL1 ChIP-Seq peaks genomic location were analyzed using a chi-square test (Fig 1G), and differences in the number of CLOCK:BMAL1 peaks per target genes (S5A Fig) were analyzed by a Fisher’s exact test. Differences were considered significant when p < 0.05.

## Supporting information

S1 Fig Assignment of CLOCK:BMAL1 DNA binding sites to their target gene transcriptional output in the mouse liver. Flowchart illustrating the procedure used to identify CLOCK:BMAL1 target genes and to determine their transcriptional output in the mouse liver. See methods section for details. Briefly, publicly available lists of CLOCK and BMAL1 DNA binding sites [from 3] were compared and the overlapping CLOCK and BMAL1 peaks were identified as CLOCK:BMAL1 DNA binding sites (BMAL1 peak coordinates were kept for downstream analysis). Of the 3217 identified CLOCK:BMAL1 peaks, 2458 were assigned to a target gene (peak located by HOMER software between -10kb of a gene transcription start site and +1kb of a gene transcription termination site). The remaining 759 peaks were listed as intergenic. The list of 2458 CLOCK:BMAL1 peaks was then parsed based on their target genes transcriptional output using our publicly available Nascent-Seq analysis of rhythmic transcription in the mouse liver [28]. 329 CLOCK:BMAL1 peaks were found to target rhythmically transcribed genes in the mouse liver. Of these, 205 peaks were found to target rhythmically transcribed genes with a peak of transcription coinciding with CLOCK:BMAL1 rhythmic DNA binding (from ZT02 to ZT12; in-phase rhythmic transcriptional cyclers or Rinφ), whereas 124 peaks were targeting genes with a peak of rhythmic transcription out-of-phase with CLOCK:BMAL1 DNA binding (from ZT12 to ZT02; out-of-phase transcription cyclers or Ro/φ). A total of 916 CLOCK:BMAL1 peaks were assigned to genes exhibiting an arrhythmic nascent RNA profile. To ensure that these target genes are “true” arrhythmically expressed target genes, the list was further filtered by removing those exhibiting rhythmic mRNA expression [using the dataset from 28], resulting in a final list of 654 CLOCK:BMAL1 peaks targeting arrhythmically transcribed genes. Finally, the remaining CLOCK:BMAL1 peaks were assigned to genes expressed below the expression threshold set to determine rhythmic gene expression. Because this threshold is set to call rhythmically expressed genes with high confidence rather than calling “true” non-expressed genes, we further filtered this list of peaks by removing genes exhibiting an averaged signal greater than 1 read/bp for the 12 time points of the Nascent-Seq dataset. This filtering resulted in a list of 291 CLOCK:BMAL1 peaks targeting non-expressed genes. The list of the 3217 CLOCK:BMAL1 peaks parsed based on their target gene transcription, and used in our meta-analysis, is provided in S1 Table.
(TIF)

S2 Fig Effect of LD *vs*. DD lighting conditions on BMAL1 rhythmic DNA binding. Mouse liver BMAL1 ChIP-Seq datasets performed in mouse exposed to LD12:12 (Rey et al., 2011) or constant darkness (DD, Koike et al., 2012) were compared to determine if the lighting conditions (LD *vs*. DD) impact BMAL1 rhythmic DNA binding phase and signal. A. Correlation between BMAL1 ChIP-Seq signal in LD and DD for each of the 4 CLOCK:BMAL1 transcriptional output categories (rhythmic-in-phase (Rinφ, red); rhythmic out-of-phase (Ro/φ, orange); arrhythmic (AR, black); and non expressed (NE, grey) target genes). Peaks targeting core clock genes are depicted with an open circle. B. Correlation between the phase of BMAL1 DNA binding in LD and DD for each of the 4 CLOCK:BMAL1 transcriptional output categories. C. Correlation between the phase of BMAL1 DNA binding in LD and DD for all 3217 CLOCK:BMAL1 ChIP-Seq peaks from the Koike et al., 2012 dataset (see methods section for details, and S1 Table). ChIP-Seq peaks were classified based on BMAL1 ChIP-Seq signal from Koike et al., 2012, and divided into 4 equal size quartiles. Peaks with higher ChIP-Seq signal display a better phase correlation in BMAL1 rhythmic DNA binding.
(TIF)

S3 Fig Analysis of BMAL1 and CLOCK ChIP-Seq signal based on the phase of target gene transcription. A. Analysis of BMAL1 ChIP-Seq signal from Koike et al. (2012) at CLOCK:BMAL1 peaks targeting rhythmically transcribed genes (RG), arrhythmically transcribed genes (AR) or not transcribed genes (NE). Peaks targeting rhythmic targets are binned in 5 groups of equal size for either all peaks (n = 329; groups RG1 to RG5), or those targeting non-core clock genes only (n = 307; groups RG1’ to RG5’). Data are represented as boxplots for each group and time points, and the thick line displays CLOCK:BMAL1 DNA binding rhythm based on the median of ChIP-Seq signal. Statistical analysis was performed by Kruskal-Wallis non-parametric test, and pair-wise post-hoc analyses are displayed for each of the six time points using color-coding of the p-values. B. Phases of nascent RNA expression of rhythmically transcribed CLOCK:BMAL1 target genes are displayed for either all rhythmic target genes (left, groups RG1 to RG5), or only non-core clock rhythmic target genes (right, groups RG1’ to RG5’). Nascent RNA expression was retrieved from Menet et al., 2012. C. Analysis of CLOCK ChIP-Seq signal from Koike et al. (2012) at CLOCK:BMAL1 peaks was performed as for BMAL1 ChIP-Seq signal in A. D. Nascent RNA expression of rhythmically transcribed CLOCK:BMAL1 is displayed for either all rhythmic targets (groups RG1 to RG5), or for non-core clock target genes (groups RG1’ to RG5’), as well was for arrhythmically transcribed target genes (AR), or non-expressed target genes (NE). Groups with different letters are significantly different (Kruskal-Wallis test; p < 0.05).
(TIF)

S4 Fig Correlation analysis between CLOCK:BMAL1 ChIP-Seq signal and CLOCK:BMAL1 target genes nascent RNA expression. A. Correlation between BMAL1 and CLOCK ChIP-Seq signal at CLOCK:BMAL1 ChIP-Seq peaks in the mouse liver from Koike et al., 2012 datasets, parsed based on the transcriptional output of CLOCK:BMAL1 target genes (in-phase transcriptional cyclers, Rinφ, red; out-of-phase transcriptional cyclers, Ro/φ, orange, arrhythmically transcribed target genes, AR, black; not transcribed target genes, NE, grey; see text for details). B. Correlation between BMAL1 (top) and CLOCK (bottom) ChIP-Seq signal and the phase of nascent RNA expression of rhythmic CLOCK:BMAL1 target genes in the mouse liver (Nascent-Seq data from Menet et al., 2012). The dash lines depict the cut-offs used to partition the in-phase cyclers (Rinφ; from ZT02 to ZT12) to the out-of-phase cyclers (Ro/φ; from ZT12 to ZT02). Distinction is made between CLOCK:BMAL1 peaks targeting core clock genes (*Per1*, *Per2*, *Cry2*, *Dbp*, *Rev-erbα*, and *Rev-erbβ*; circles filled in red), extended core clock genes (*Tef*, *Hlf*, *Gm129*, and *Rorγ*; circles filled in orange), to those targeting clock-controlled genes (filled in blue and green for BMAL1 and CLOCK, respectively). CLOCK:BMAL1 peaks targeting arrhythmically transcribed genes (circles filled in black) and non expressed genes (circles filled in grey) are shown for comparison. C. Correlation between the phase of BMAL1 (top) or CLOCK (bottom) DNA binding and the phase of transcription of rhythmically transcribed CLOCK:BMAL1 target genes in the mouse liver. D. Correlation between BMAL1 ChIP-Seq signal and nascent RNA expression levels of CLOCK:BMAL1 target genes in the mouse liver, parsed based on the transcriptional output of CLOCK:BMAL1 target genes.
(TIF)

S5 Fig Contribution of CLOCK:BMAL1 peaks targeting the same genes to CLOCK:BMAL1 ChIP-Seq signal and CLOCK:BMAL1 target gene nascent RNA expression. A. The number of CLOCK:BMAL1 target genes is displayed based on the number of CLOCK:BMAL1 ChIP-Seq peaks for each of the 4 categories of CLOCK:BMAL1 transcriptional output (in-phase transcriptional cyclers, Rinφ, red; out-of-phase transcriptional cyclers, Ro/φ, orange, arrhythmically transcribed target genes, AR, black; not transcribed target genes, NE, grey; see text for details). Top table: all CLOCK:BMAL1 target genes; Middle table: target genes without core clock genes (*Per1*, *Per2*, *Cry2*, *Dbp*, *Rev-erbα*, and *Rev-erbβ*); Bottom table: CLOCK:BMAL1 target genes without core clock genes (*Per1*, *Per2*, *Cry2*, *Dbp*, *Rev-erbα*, and *Rev-erbβ*) and other associated clock genes (*Tef*, *Hlf*, *Gm129*, and *Rorγ*). Yellow boxes indicate the location of clock genes within the table. The distribution of the number of CLOCK:BMAL1 ChIP-Seq peaks per gene is also displayed as a pie chart for all CLOCK:BMAL1 peaks. Groups with different letters are significantly different (Fischer's exact test (two-sided test); p < 0.05). B, C. BMAL1 (B) and CLOCK (C) ChIP-Seq signal at CLOCK:BMAL1 ChIP-Seq peaks (from Koike et al., 2012) is displayed for each of CLOCK:BMAL1 transcriptional output category. In this analysis, ChIP-Seq signal at CLOCK:BMAL1 peaks targeting the same gene was summed up (see panel A for the number of genes with multiple peaks for each category). Groups with different letters are significantly different (Kruskal-Wallis test; p < 0.05). D. Nascent RNA expression of CLOCK:BMAL1 target genes parsed based on CLOCK:BMAL1 target gene transcription, for all CLOCK:BMAL1 targets (left), target genes without core clock genes (middle), and target genes without core clock genes and other associated clock genes (*Tef*, *Hlf*, *Gm129*, and *Rorγ*). Groups with different letters are significantly different (Kruskal-Wallis test; p < 0.05).
(TIF)

S6 Fig Recruitment of PERs and CRYs to CLOCK:BMAL1 peaks does not correlate with the heterogeneous CLOCK:BMAL1 transcriptional output. A. (*Left*) Circadian rhythm of BMAL1 and CLOCK ChIP-Seq signal in the mouse liver at CLOCK:BMAL1 DNA binding sites for each of the 4 CLOCK:BMAL1 transcriptional output groups. (*Right*) Distribution of BMAL1 and CLOCK ChIP-Seq signal for each of the 4 CLOCK:BMAL1 transcriptional output groups at the time of maximal DNA binding (CT04 for BMAL1 and CT08 for CLOCK). B. (*Left*) Circadian rhythm of PER1, PER2, CRY1, and CRY2 ChIP-Seq signal in the mouse liver at CLOCK:BMAL1 DNA binding sites for each of the 4 CLOCK:BMAL1 transcriptional output groups. (*Right*) Distribution of PER1, PER2, CRY1, and CRY2 ChIP-Seq signal for each of the 4 CLOCK:BMAL1 transcriptional output group at the time of maximal DNA binding (CT16 for PER1 and PER2, CT04 for CRY1 and CT12 for CRY2. For both A and B panels, datasets were retrieved from Koike et al., 2012 and re-analyzed (see methods section for more details). Values correspond to the ChIP-Seq signal median for each group. To improve visualization, CT0 ChIP-Seq values were repeated at CT24. Groups with different letters are significantly different (Kruskal-Wallis test; p < 0.05).
(TIF)

S7 Fig Rhythmic nucleosome signal at CLOCK:BMAL1 DNA binding sites. A-D: Nucleosome signal was retrieved from mouse liver MNase-Seq datasets (Menet et al., 2014), which consists of 6 time points each separated by 4 hours with n = 4 mice for each time point. Each graph displays a 6-time points rhythm of nucleosome signal at CLOCK:BMAL1 binding sites (calculated at CLOCK:BMAL1 peak center ± 75 bp for each peak), displayed as the average ± s.e.m. of the signal (n = 4) calculated for each mouse and for each transcriptional output category: (A) Rhythmic-in-phase (Rinφ, red); (B) Rhythmic out-of-phase (Ro/φ, orange); (C) arrhythmic (AR, black); (D) non expressed (NE, grey) target genes). The phase of rhythm (average ± s.e.m. from 4 independent rhythm, calculated by Fourier transform) is indicated in the bottom right. Each rhythm is double-plotted for better visualization. For both Rinφ and Ro/φ groups, the nucleosome rhythm is calculated at all CLOCK:BMAL1 peaks targeting rhythmic target genes (left), or only at peaks targeting rhythmic non core clock genes (removal of nucleosome signal at peaks targeting *Cry2*, *Dbp*, *Rev-erbα*, and *Rev-erbβ* for the Rinφ group, and of the peaks targeting *Per1* and *Per2* for the Ro/φ group. E: maximal and minimal nucleosome signal from the 6-time points rhythms for each of the CLOCK:BMAL1 transcriptional output groups. Groups with different letters are significantly different (2-way ANOVA; p < 0.05).
(TIF)

S8 Fig CLOCK:BMAL1 does not directly promote H3K27ac post-translational modification. A. Circadian rhythm of H3K27ac ChIP-Seq signal in the mouse liver at CLOCK:BMAL1 DNA binding sites (*Left; blue background*) or at non-CLOCK:BMAL1 enhancers located in CLOCK:BMAL1 target genes (*Right; green background*) for each of the 4 CLOCK:BMAL1 transcriptional output groups: rhythmic-in-phase (Rinφ, red); rhythmic out-of-phase (Ro/φ, orange); arrhythmic (AR, black); and non expressed (NE, grey) target genes. Datasets were retrieved from Koike et al., 2012 (top) or Vollmers et al., 2012 (bottom) and re-analyzed (see methods section for more details). Values correspond to the ChIP-Seq signal median for each group, and were calculated for each CLOCK:BMAL1 peak as the average of reads/bp at CLOCK:BMAL1 DNA binding sites center ± 1 kb normalized to one million sequencing reads. For each dataset, H3K27ac ChIP-Seq signal was further normalized by mean normalization to account for the differences in ChIP-Seq efficiency between each sequencing sample (bottom graphs for each datasets). This normalization assumes that the overall genome-wide levels of H3K27ac are constant at any time in the mouse liver. To this end, we normalized H3K27ac ChIP-Seq signal for each peak to the averaged H3K27ac signal calculated at the top 40,000 DNase hypersensitive sites for each time point Graphs are double-plotted to improve visualization. B. Rhythm of enhancer RNA (eRNA) signal in the mouse liver at enhancers targeting a CLOCK:BMAL1 target gene and that do not harbor a CLOCK:BMAL1 DNA binding site (calculated at CLOCK:BMAL1 peak center ± 500 bp) for each of the 4 CLOCK:BMAL1 transcriptional output group. Datasets were retrieved from Fang et al., 2014 [36] and re-analyzed (see methods section for details). Values correspond to the eRNA signal median for each group. Each rhythm is double-plotted for better visualization. C. Rhythm of RNA Polymerase II ChIP-Seq signal in the mouse liver at CLOCK:BMAL1 enhancers (top) and enhancers targeting a CLOCK:BMAL1 target gene but without a CLOCK:BMAL1 DNA binding site for each of the 4 CLOCK:BMAL1 transcriptional output group. Datasets were retrieved from Le Martelot et al., 2012 [22], re-analyzed and normalized by a ranking analysis (see methods section for details). Values correspond to Pol II ChIP-Seq signal median for each group. Each rhythm is double-plotted for better visualization.
(TIF)

S9 Fig Transcription factor DNA binding motif analysis at CLOCK:BMAL1 enhancers and based on CLOCK:BMAL1 transcriptional output. Enrichment for transcription factor DNA binding motifs (calculated using the HOMER software suite) at CLOCK:BMAL1 DNA binding sites for each of the four CLOCK:BMAL1 transcriptional output categories: rhythmic-in-phase (Rinφ, red); rhythmic out-of-phase (Ro/φ, orange); arrhythmic (AR, black); and non expressed (NE, grey) target genes. Enrichment is reported as the ratio between the calculated enrichment over the calculated background. * q < 0.05 (Benjamini-Hochberg procedure). Motif enrichment is shown for: CLOCK:BMAL1 (A); tissue-specific transcription factors (B); as well as ubiquitous transcription factors for which the motif is enriched for all 4 output groups (C) or specific group(s) (D).
(TIF)

S10 Fig Transcription factor ChIP-Seq signal at CLOCK:BMAL1 enhancers and based on CLOCK:BMAL1 transcriptional output. Mouse liver ChIP-Seq signal of tissue-specific transcription factors (A), ubiquitous transcription factors (B, C), and transcriptional co-activators / RNA Polymerase II at ZT10 (D) at CLOCK:BMAL1 DNA binding sites (peak center ± 250bp) for each of the transcriptional output categories. ChIP-Seq signal is represented for each output group based on its distribution (every decile). Groups with different letters are significantly different (Kruskal-Wallis test; p < 0.05).
(TIF)

S11 Fig Analysis of nucleosome signal over the 24-hr day at the DNA binding sites of two tissue-specific TF and two ubiquitous TF in the mouse liver. Nucleosome signal at four TF DNA binding sites was retrieved from mouse liver MNase-Seq datasets (Menet et al., 2014), which consists of 6 time points each separated by 4 hours with n = 4 mice for each time point. MNase-Seq data are displayed for two tissue-specific TFs: FOXA2 (A) and HNF6 (B), and two ubiquitous TFs: REV-ERBα (C) and ERα (D). Nucleosome signal was calculated at TF peak center ± 75 bp for each peak and at each TF ChIP-Seq peak coordinate, and is displayed as the average ± s.e.m of the signal (n = 4) calculated for each mouse. Each rhythm is double-plotted for better visualization. *(Left*, *A-D)* Nucleosome signal at TF ChIP-Seq peaks harboring a CLOCK:BMAL1 peak (blue), or at the top 10,000 TF ChIP-Seq peaks that do not harbor a CLOCK:BMAL1 peak (green). *(Right*, *A’-D’)* Nucleosome signal at TF ChIP-Seq peaks harboring a CLOCK:BMAL1 peak, and parsed based on CLOCK:BMAL1 transcriptional output: Rhythmic-in-phase (Rinφ, red), Rhythmic out-of-phase (Ro/φ, orange), arrhythmic (AR, black), and non expressed (NE, grey).
(TIF)

S12 Fig Additional examples of CLOCK:BMAL1 target genes exhibiting changes in expression under fasting condition in the mouse liver. Rhythm of mRNA expression in the liver of mice fed ad libitum (blue) or fasted for at least 22 hours (orange). Data were retrieved from a public dataset (Vollmers et al., 2009). Mouse liver mRNA expression is displayed for CLOCK:BMAL1 target genes that are rhythmically expressed in the liver of mice fed *ad libitum*, and that exhibit under fasting condition a decrease in the rhythm amplitude (first column), no change (second column) or an increase in the rhythm amplitude (third column). Mouse liver mRNA expression is also displayed for CLOCK:BMAL1 target genes that are arrhythmically expressed in the liver of mice fed *ad libitum*, and that exhibit rhythmic expression under fasting condition.
(TIF)

S13 Fig Effect of high-fat diet on CLOCK:BMAL1 target gene expression. A-D. Six-time point rhythm of liver mRNA expression in mice fed with normal chow (black) or high fat diet (blue). Values were retrieved from a public dataset [57] and correspond to the average ± s.e.m. of 3 independent samples. Mouse liver mRNA expression is displayed for *Clock* and *Bmal1* (A), and some CLOCK:BMAL1 target genes that are rhythmic under both normal chow and high fat diet (B); normal chow only (C); and high fat diet only (D).
(TIF)

S14 Fig Analysis of nucleosome signal, enhancer activity, and TF DNA binding at cis-regulatory regions targeting non-CLOCK:BMAL1 target genes. Analysis of a random set of genes not targeted by CLOCK:BMAL1 and transcribed similarly to the 4 CLOCK:BMAL1 transcriptional output groups (n = 125 genes for each group) was performed to determine the extent to which the findings reported in the manuscript are specific to CLOCK:BMAL1. The same criteria as those used for the characterization of CLOCK:BMAL1 transcriptional output were used, and groups are thus similarly referred as Rhythmic-in-phase (Rinφ, dark blue), Rhythmic out-of-phase (Ro/φ, light blue), arrhythmic (AR, black), and non expressed (NE, grey). Cis-regulatory regions targeting the randomly selected control genes are defined as DNase I hypersensitive sites (DHS) located within -10kb of the TSS to +1kb of the TTS (similarly to what was done for CLOCK:BMAL1 target genes). Analysis of these DHS suggests that many of our findings are specific to CLOCK:BMAL1 A. Heatmap displaying nascent RNA expression of the random set of genes and parsed based on the transcriptional output. Nascent-Seq signal was ordered based on the phase of nascent RNA oscillations for the in-phase and out-of-phase transcriptional cyclers. Ordering of arrhythmically transcribed genes is based on the peak time of maximal expression; the lack of a distinctive 24-hr rhythm profile of nascent RNA expression over the 48-hr time-scale is indicative of arrhythmic transcription. NE genes are not displayed due to the lack of expression. B. Average nascent RNA expression level for the 4 control groups. C-D. BMAL1 (C) and CLOCK (D) ChIP-Seq signal at DNase I hypersensitive sites (DHS) targeting the randomly selected control genes. ChIP-Seq signal for CLOCK:BMAL1 target genes is provided for comparison. E. Nucleosome rhythm at DHS targeting the randomly selected control genes (similar to Fig 3A–3D). F-H. H3K27ac, Pol II and eRNA expression at DHS targeting the randomly selected control genes (similar to Figs 3F, S8C and S3G, respectively). I-K. ts-TF (I), u-TF (J), and p300 and Pol II (K) ChIP-Seq signal at DHS targeting the randomly selected control genes (similar to Fig 4D–4F).
(TIF)

S15 Fig The enrichment of CTCF (CCCTC-binding factor) at CLOCK:BMAL1 enhancers targeting non-expressed genes may underscore the role of long-range chromatin interactions between CLOCK:BMAL1 enhancers and its target genes. Visualization file of ChIP-Seq signal at two CLOCK:BMAL1 enhancers (yellow boxes) targeting non expressed genes (Top: *Lif* and Bottom: *Adc*), and exhibiting significant CTCF ChIP-Seq signal. Arrows with question marks indicate a potential CTCF-mediated long-range chromatin interaction that would enable CLOCK:BMAL1 to regulate the rhythmic transcription of genes located more than 50kb away from its enhancer.
(TIF)

S1 Table List of the CLOCK:BMAL1 peaks parsed based on the transcriptional output. This table contains the list of the 3217 CLOCK:BMAL1 peaks that was used in this study, along with their genomic localization (mm10 version), their target gene, and their parsing into specific transcriptional output categories.
(TXT)

S2 Table Motif analysis at CLOCK:BMAL1 DNA binding sites based on the transcriptional output. Outcome of the motif analysis performed by the HOMER software at CLOCK:BMAL1 DNA binding sites parsed based on their transcriptional output.
(XLSX)

S3 Table TF ChIP-Seq signal at CLOCK:BMAL1 peaks parsed based on the transcriptional output. This table contains the ChIP-Seq signal for several TFs at the 1,274 CLOCK:BMAL1 peaks that were assigned to a CLOCK:BMAL1 transcriptional output category used in this study.
(XLSX)

S4 Table Genomic coordinates of control genes not targeted by CLOCK:BMAL1 and their DNase hypersensitive sites This table contains the lists of genes not targeted by CLOCK:BMAL1 that were randomly selected and used to determine if the mechanisms underlying CLOCK:BMAL1-mediated transcription are specific (S14 Fig). The genomic coordinates of these control genes correspond to -10kb from the TSS to +1kb from the TTS (mm10 version). The table also contains the genomic location of the top 40,000 DNase hypersensitive sites (given size) located within these control genes (-10kb from the TSS to +1kb from the TTS), and that were used in our analysis.
(XLSX)
