# Supplementary figures and images for: Regulation of circadian clock transcriptional output by CLOCK:BMAL1
Source: PLoS Genet. 2018 Jan 4;14(1):e1007156. doi: 10.1371/journal.pgen.1007156 (PMC5771620; doi:10.1371/journal.pgen.1007156)

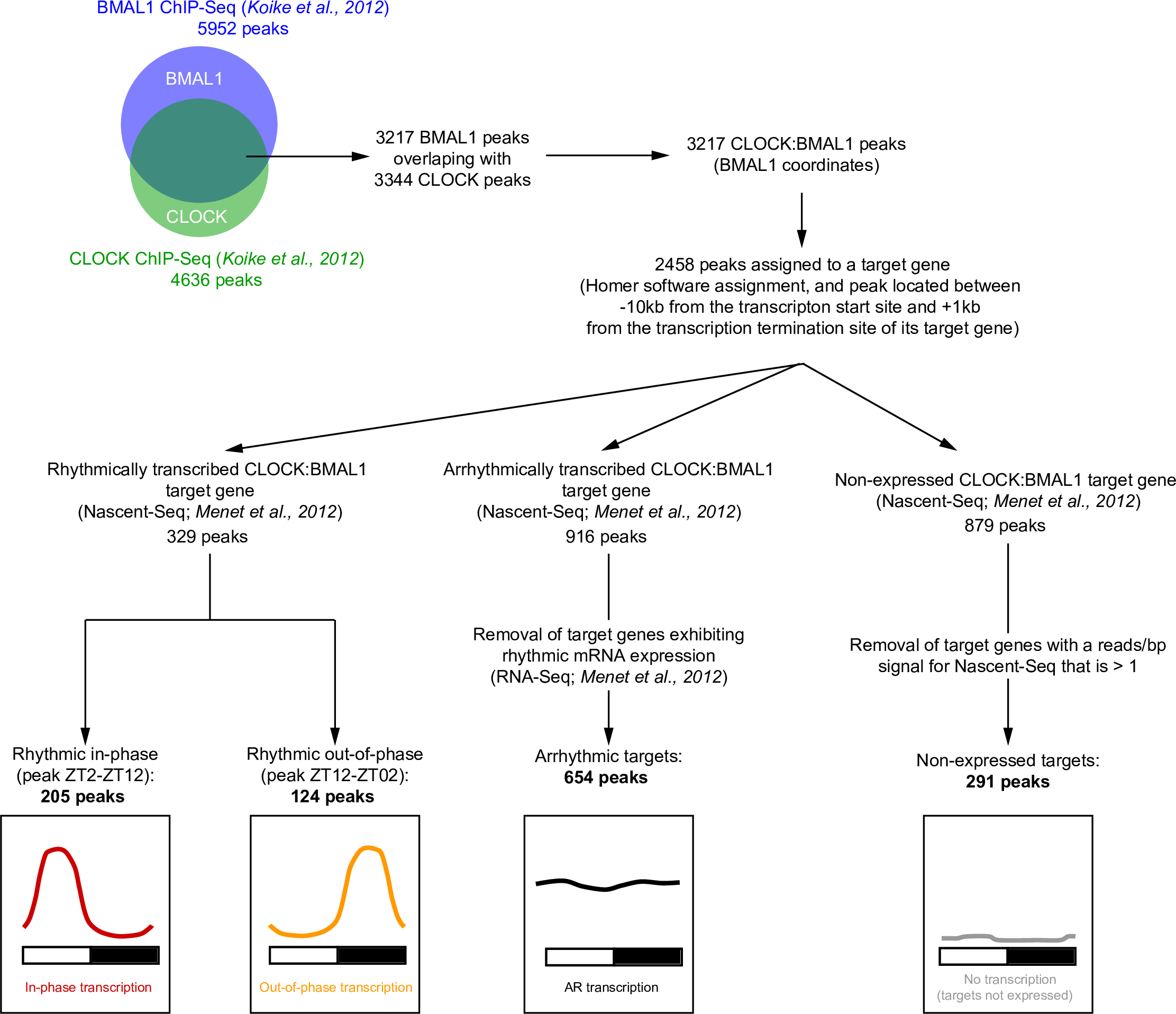

Supplement: S1 Fig — Flowchart illustrating the procedure used to identify CLOCK:BMAL1 target genes and to determine their transcriptional output in the mouse liver. See methods section for details. Briefly, publicly available lists of CLOCK and BMAL1 DNA binding sites [from 3] were compared and the overlapping CLOCK and BMAL1 peaks were identified as CLOCK:BMAL1 DNA binding sites (BMAL1 peak coordinates were kept for downstream analysis). Of the 3217 identified CLOCK:BMAL1 peaks, 2458 were assigned to a target gene (peak located by HOMER software between -10kb of a gene transcription start site and +1kb of a gene transcription termination site). The remaining 759 peaks were listed as intergenic. The list of 2458 CLOCK:BMAL1 peaks was then parsed based on their target genes transcriptional output using our publicly available Nascent-Seq analysis of rhythmic transcription in the mouse liver [28]. 329 CLOCK:BMAL1 peaks were found to target rhythmically transcribed genes in the mouse liver. Of these, 205 peaks were found to target rhythmically transcribed genes with a peak of transcription coinciding with CLOCK:BMAL1 rhythmic DNA binding (from ZT02 to ZT12; in-phase rhythmic transcriptional cyclers or Rinφ), whereas 124 peaks were targeting genes with a peak of rhythmic transcription out-of-phase with CLOCK:BMAL1 DNA binding (from ZT12 to ZT02; out-of-phase transcription cyclers or Ro/φ). A total of 916 CLOCK:BMAL1 peaks were assigned to genes exhibiting an arrhythmic nascent RNA profile. To ensure that these target genes are “true” arrhythmically expressed target genes, the list was further filtered by removing those exhibiting rhythmic mRNA expression [using the dataset from 28], resulting in a final list of 654 CLOCK:BMAL1 peaks targeting arrhythmically transcribed genes. Finally, the remaining CLOCK:BMAL1 peaks were assigned to genes expressed below the expression threshold set to determine rhythmic gene expression. Because this threshold is set to call rhythmically expressed genes wit [file pgen.1007156.s001.tif]

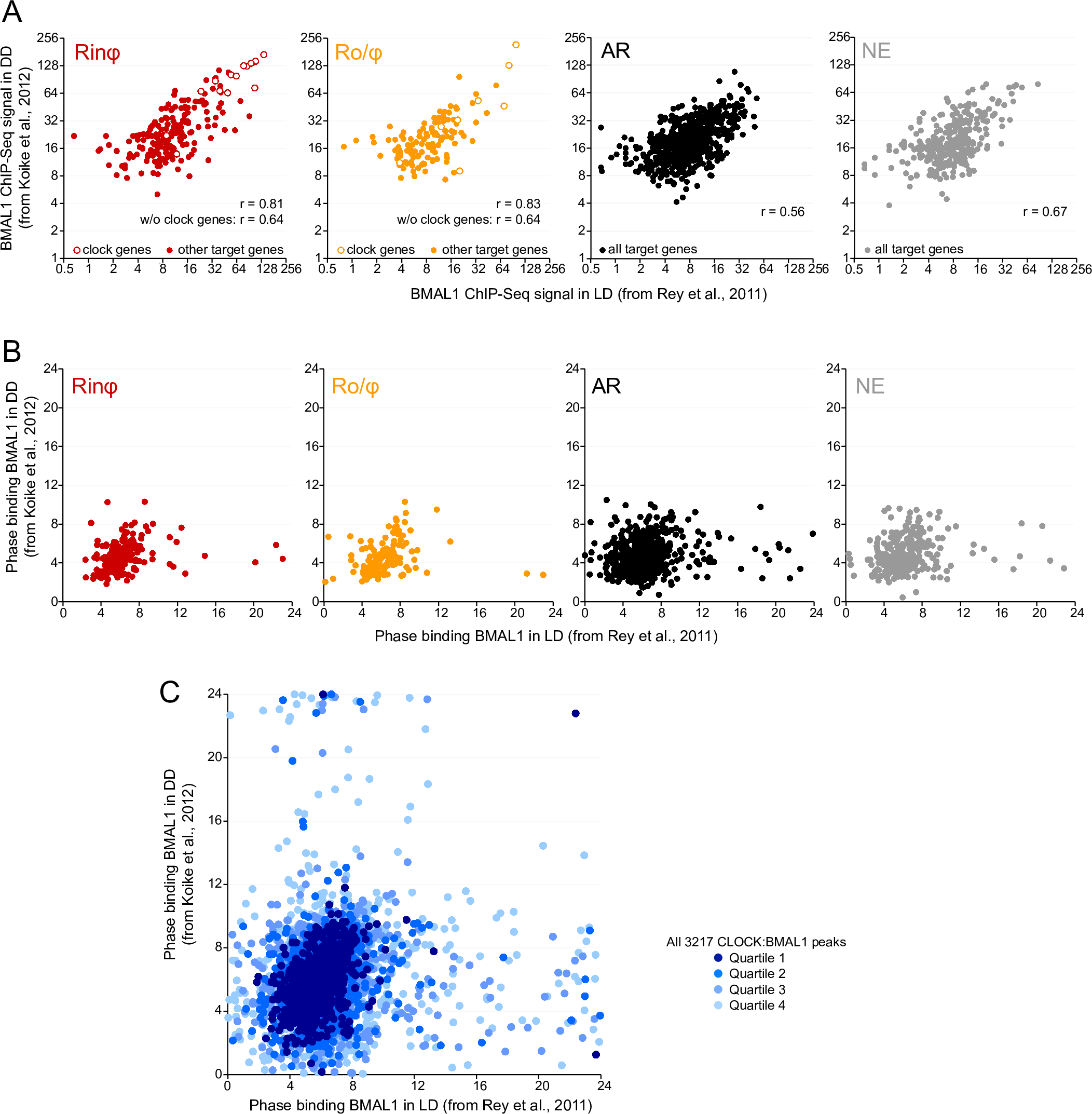

Supplement: S2 Fig — Mouse liver BMAL1 ChIP-Seq datasets performed in mouse exposed to LD12:12 (Rey et al., 2011) or constant darkness (DD, Koike et al., 2012) were compared to determine if the lighting conditions (LD vs. DD) impact BMAL1 rhythmic DNA binding phase and signal. A. Correlation between BMAL1 ChIP-Seq signal in LD and DD for each of the 4 CLOCK:BMAL1 transcriptional output categories (rhythmic-in-phase (Rinφ, red); rhythmic out-of-phase (Ro/φ, orange); arrhythmic (AR, black); and non expressed (NE, grey) target genes). Peaks targeting core clock genes are depicted with an open circle. B. Correlation between the phase of BMAL1 DNA binding in LD and DD for each of the 4 CLOCK:BMAL1 transcriptional output categories. C. Correlation between the phase of BMAL1 DNA binding in LD and DD for all 3217 CLOCK:BMAL1 ChIP-Seq peaks from the Koike et al., 2012 dataset (see methods section for details, and S1 Table). ChIP-Seq peaks were classified based on BMAL1 ChIP-Seq signal from Koike et al., 2012, and divided into 4 equal size quartiles. Peaks with higher ChIP-Seq signal display a better phase correlation in BMAL1 rhythmic DNA binding. (TIF) [file pgen.1007156.s002.tif]

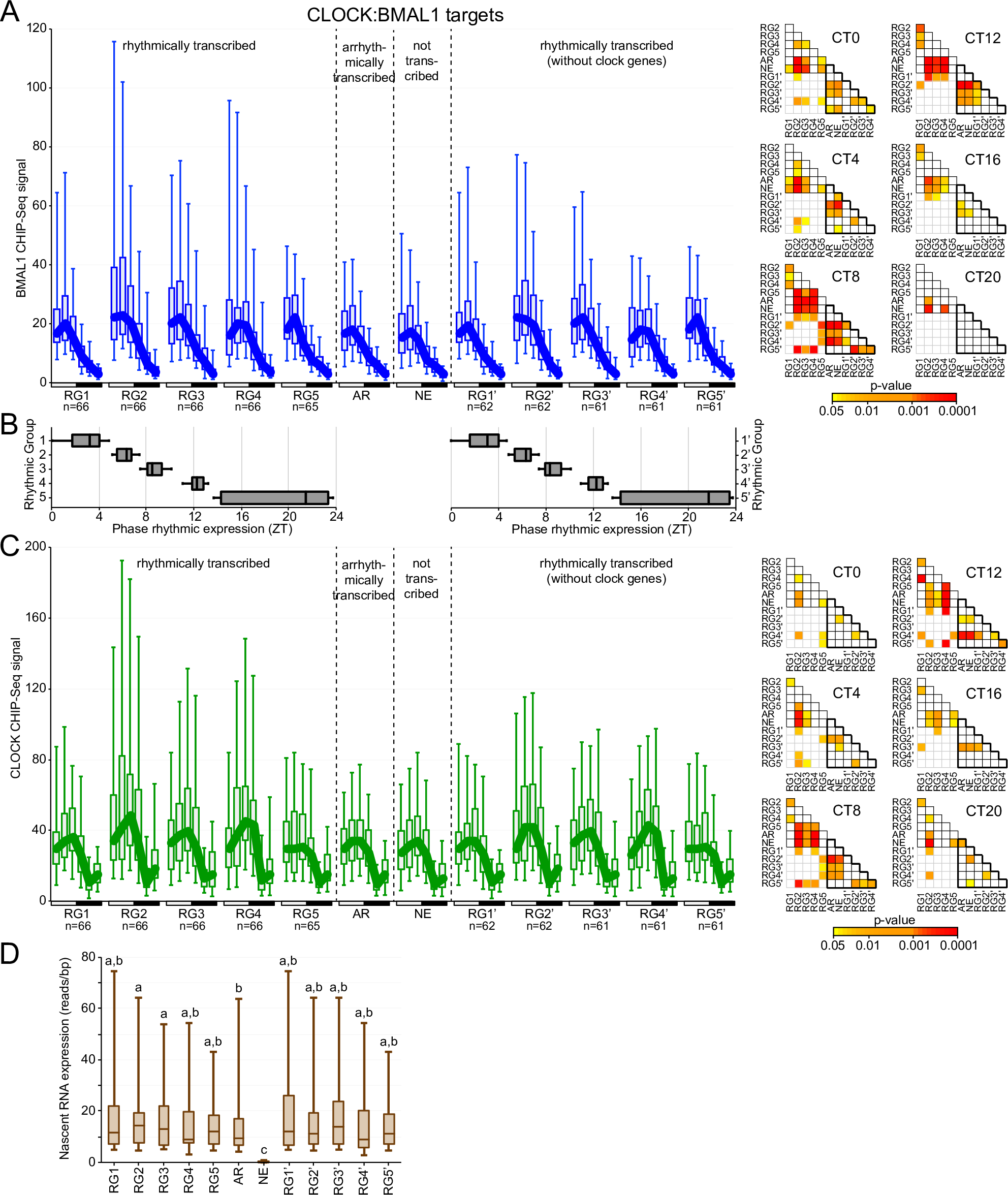

Supplement: S3 Fig — A. Analysis of BMAL1 ChIP-Seq signal from Koike et al. (2012) at CLOCK:BMAL1 peaks targeting rhythmically transcribed genes (RG), arrhythmically transcribed genes (AR) or not transcribed genes (NE). Peaks targeting rhythmic targets are binned in 5 groups of equal size for either all peaks (n = 329; groups RG1 to RG5), or those targeting non-core clock genes only (n = 307; groups RG1’ to RG5’). Data are represented as boxplots for each group and time points, and the thick line displays CLOCK:BMAL1 DNA binding rhythm based on the median of ChIP-Seq signal. Statistical analysis was performed by Kruskal-Wallis non-parametric test, and pair-wise post-hoc analyses are displayed for each of the six time points using color-coding of the p-values. B. Phases of nascent RNA expression of rhythmically transcribed CLOCK:BMAL1 target genes are displayed for either all rhythmic target genes (left, groups RG1 to RG5), or only non-core clock rhythmic target genes (right, groups RG1’ to RG5’). Nascent RNA expression was retrieved from Menet et al., 2012. C. Analysis of CLOCK ChIP-Seq signal from Koike et al. (2012) at CLOCK:BMAL1 peaks was performed as for BMAL1 ChIP-Seq signal in A. D. Nascent RNA expression of rhythmically transcribed CLOCK:BMAL1 is displayed for either all rhythmic targets (groups RG1 to RG5), or for non-core clock target genes (groups RG1’ to RG5’), as well was for arrhythmically transcribed target genes (AR), or non-expressed target genes (NE). Groups with different letters are significantly different (Kruskal-Wallis test; p < 0.05). (TIF) [file pgen.1007156.s003.tif]

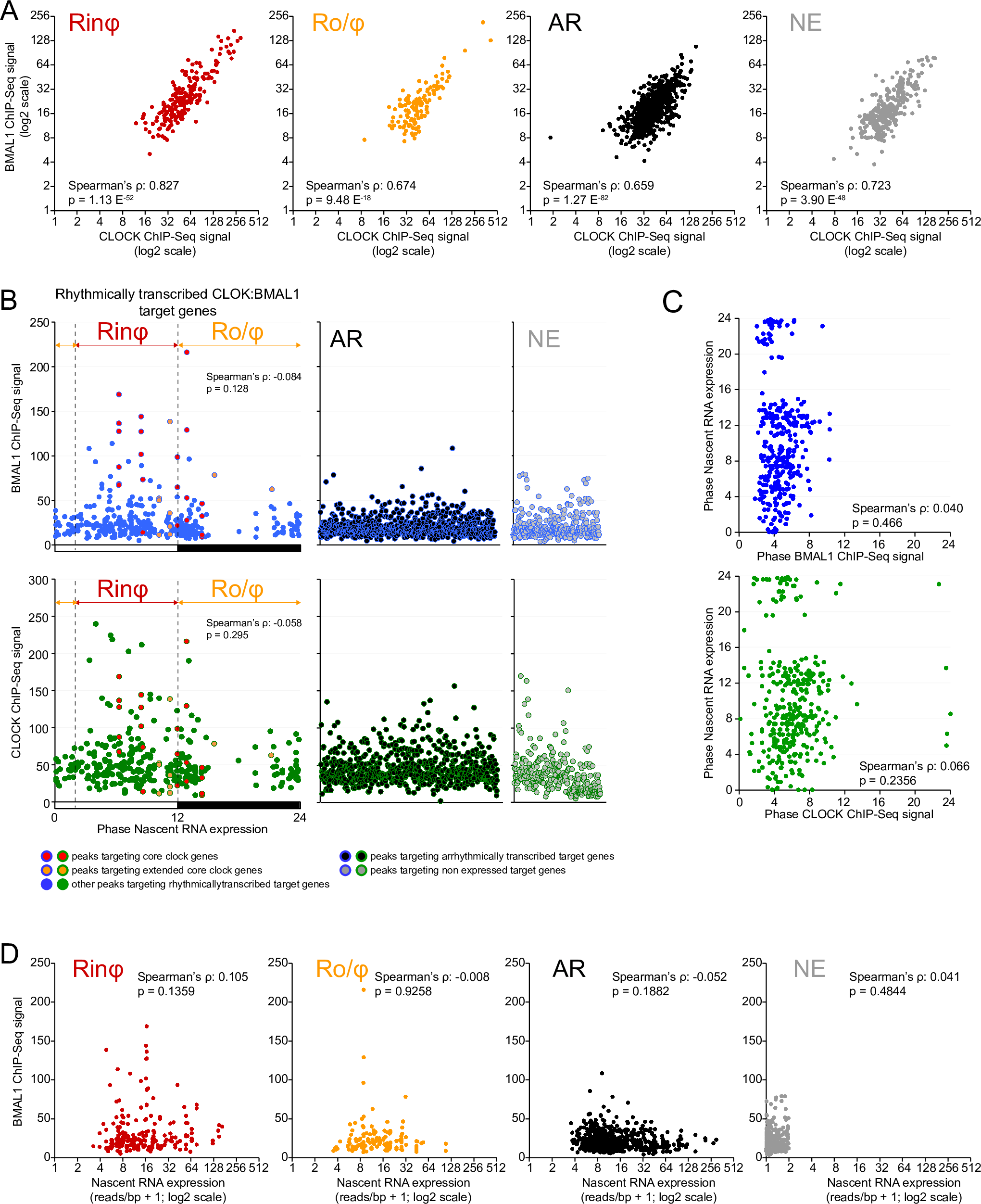

Supplement: S4 Fig — A. Correlation between BMAL1 and CLOCK ChIP-Seq signal at CLOCK:BMAL1 ChIP-Seq peaks in the mouse liver from Koike et al., 2012 datasets, parsed based on the transcriptional output of CLOCK:BMAL1 target genes (in-phase transcriptional cyclers, Rinφ, red; out-of-phase transcriptional cyclers, Ro/φ, orange, arrhythmically transcribed target genes, AR, black; not transcribed target genes, NE, grey; see text for details). B. Correlation between BMAL1 (top) and CLOCK (bottom) ChIP-Seq signal and the phase of nascent RNA expression of rhythmic CLOCK:BMAL1 target genes in the mouse liver (Nascent-Seq data from Menet et al., 2012). The dash lines depict the cut-offs used to partition the in-phase cyclers (Rinφ; from ZT02 to ZT12) to the out-of-phase cyclers (Ro/φ; from ZT12 to ZT02). Distinction is made between CLOCK:BMAL1 peaks targeting core clock genes (Per1, Per2, Cry2, Dbp, Rev-erbα, and Rev-erbβ; circles filled in red), extended core clock genes (Tef, Hlf, Gm129, and Rorγ; circles filled in orange), to those targeting clock-controlled genes (filled in blue and green for BMAL1 and CLOCK, respectively). CLOCK:BMAL1 peaks targeting arrhythmically transcribed genes (circles filled in black) and non expressed genes (circles filled in grey) are shown for comparison. C. Correlation between the phase of BMAL1 (top) or CLOCK (bottom) DNA binding and the phase of transcription of rhythmically transcribed CLOCK:BMAL1 target genes in the mouse liver. D. Correlation between BMAL1 ChIP-Seq signal and nascent RNA expression levels of CLOCK:BMAL1 target genes in the mouse liver, parsed based on the transcriptional output of CLOCK:BMAL1 target genes. (TIF) [file pgen.1007156.s004.tif]

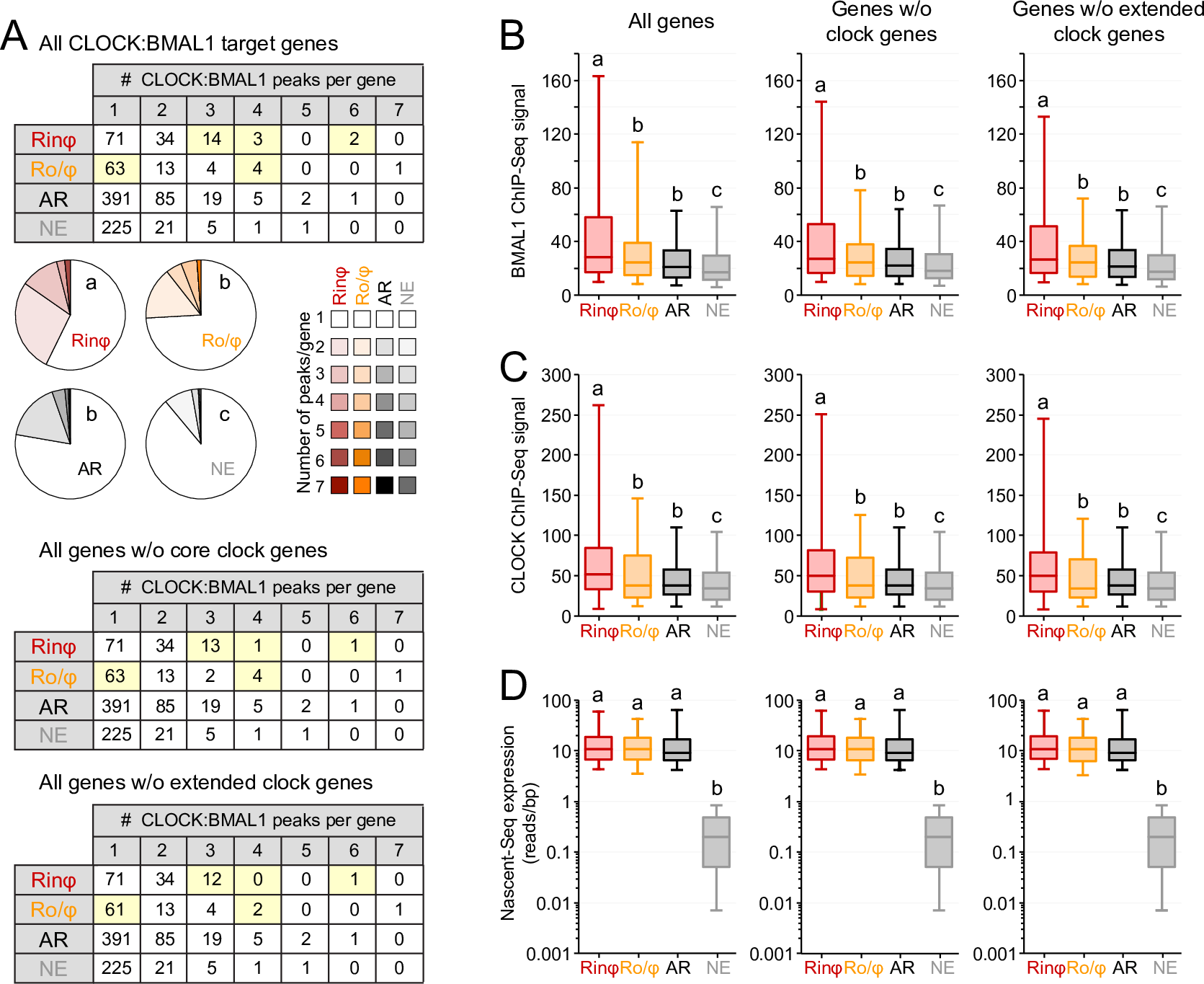

Supplement: S5 Fig — A. The number of CLOCK:BMAL1 target genes is displayed based on the number of CLOCK:BMAL1 ChIP-Seq peaks for each of the 4 categories of CLOCK:BMAL1 transcriptional output (in-phase transcriptional cyclers, Rinφ, red; out-of-phase transcriptional cyclers, Ro/φ, orange, arrhythmically transcribed target genes, AR, black; not transcribed target genes, NE, grey; see text for details). Top table: all CLOCK:BMAL1 target genes; Middle table: target genes without core clock genes (Per1, Per2, Cry2, Dbp, Rev-erbα, and Rev-erbβ); Bottom table: CLOCK:BMAL1 target genes without core clock genes (Per1, Per2, Cry2, Dbp, Rev-erbα, and Rev-erbβ) and other associated clock genes (Tef, Hlf, Gm129, and Rorγ). Yellow boxes indicate the location of clock genes within the table. The distribution of the number of CLOCK:BMAL1 ChIP-Seq peaks per gene is also displayed as a pie chart for all CLOCK:BMAL1 peaks. Groups with different letters are significantly different (Fischer's exact test (two-sided test); p < 0.05). B, C. BMAL1 (B) and CLOCK (C) ChIP-Seq signal at CLOCK:BMAL1 ChIP-Seq peaks (from Koike et al., 2012) is displayed for each of CLOCK:BMAL1 transcriptional output category. In this analysis, ChIP-Seq signal at CLOCK:BMAL1 peaks targeting the same gene was summed up (see panel A for the number of genes with multiple peaks for each category). Groups with different letters are significantly different (Kruskal-Wallis test; p < 0.05). D. Nascent RNA expression of CLOCK:BMAL1 target genes parsed based on CLOCK:BMAL1 target gene transcription, for all CLOCK:BMAL1 targets (left), target genes without core clock genes (middle), and target genes without core clock genes and other associated clock genes (Tef, Hlf, Gm129, and Rorγ). Groups with different letters are significantly different (Kruskal-Wallis test; p < 0.05). (TIF) [file pgen.1007156.s005.tif]

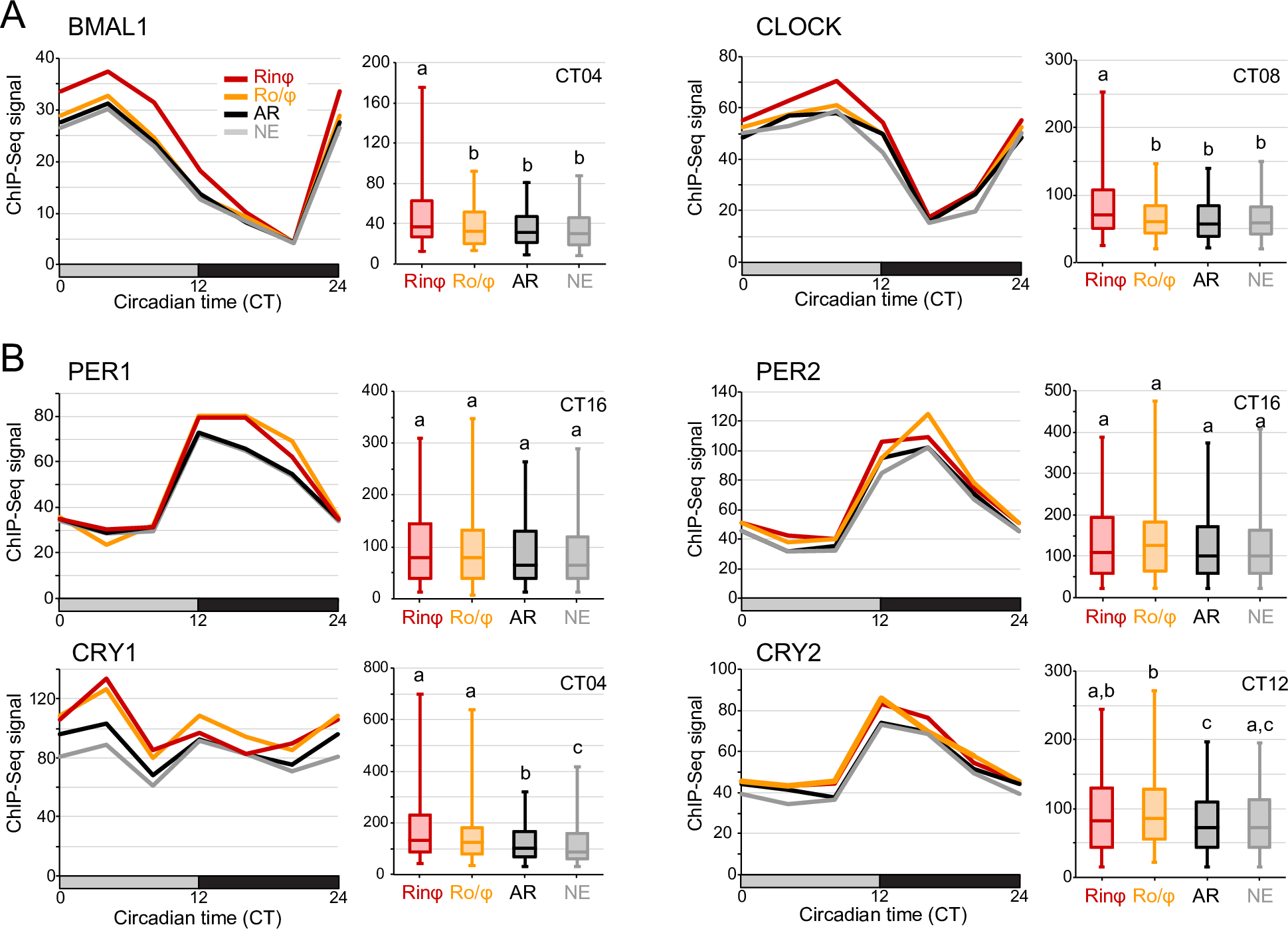

Supplement: S6 Fig — A. (Left) Circadian rhythm of BMAL1 and CLOCK ChIP-Seq signal in the mouse liver at CLOCK:BMAL1 DNA binding sites for each of the 4 CLOCK:BMAL1 transcriptional output groups. (Right) Distribution of BMAL1 and CLOCK ChIP-Seq signal for each of the 4 CLOCK:BMAL1 transcriptional output groups at the time of maximal DNA binding (CT04 for BMAL1 and CT08 for CLOCK). B. (Left) Circadian rhythm of PER1, PER2, CRY1, and CRY2 ChIP-Seq signal in the mouse liver at CLOCK:BMAL1 DNA binding sites for each of the 4 CLOCK:BMAL1 transcriptional output groups. (Right) Distribution of PER1, PER2, CRY1, and CRY2 ChIP-Seq signal for each of the 4 CLOCK:BMAL1 transcriptional output group at the time of maximal DNA binding (CT16 for PER1 and PER2, CT04 for CRY1 and CT12 for CRY2. For both A and B panels, datasets were retrieved from Koike et al., 2012 and re-analyzed (see methods section for more details). Values correspond to the ChIP-Seq signal median for each group. To improve visualization, CT0 ChIP-Seq values were repeated at CT24. Groups with different letters are significantly different (Kruskal-Wallis test; p < 0.05). (TIF) [file pgen.1007156.s006.tif]

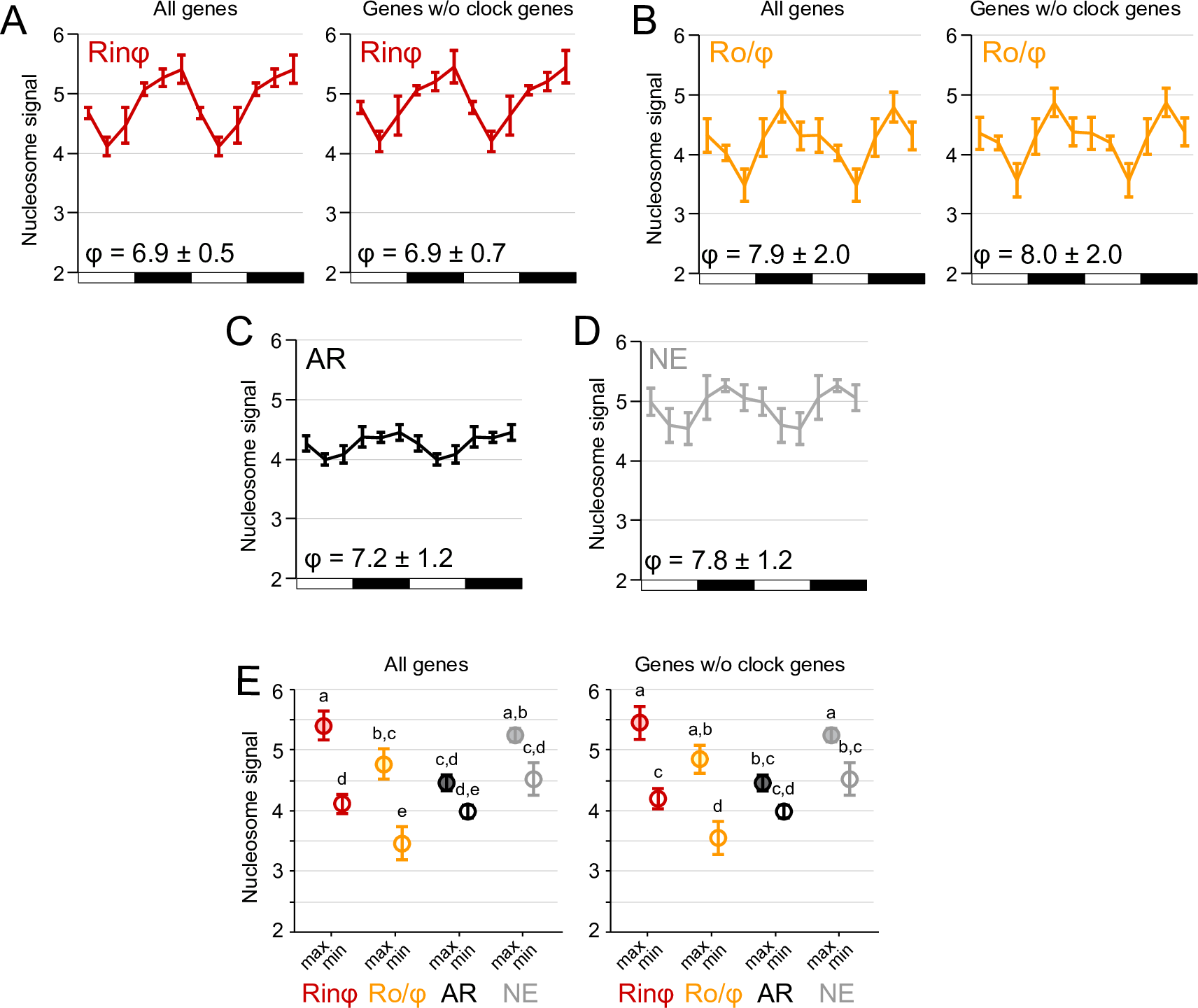

Supplement: S7 Fig — A-D: Nucleosome signal was retrieved from mouse liver MNase-Seq datasets (Menet et al., 2014), which consists of 6 time points each separated by 4 hours with n = 4 mice for each time point. Each graph displays a 6-time points rhythm of nucleosome signal at CLOCK:BMAL1 binding sites (calculated at CLOCK:BMAL1 peak center ± 75 bp for each peak), displayed as the average ± s.e.m. of the signal (n = 4) calculated for each mouse and for each transcriptional output category: (A) Rhythmic-in-phase (Rinφ, red); (B) Rhythmic out-of-phase (Ro/φ, orange); (C) arrhythmic (AR, black); (D) non expressed (NE, grey) target genes). The phase of rhythm (average ± s.e.m. from 4 independent rhythm, calculated by Fourier transform) is indicated in the bottom right. Each rhythm is double-plotted for better visualization. For both Rinφ and Ro/φ groups, the nucleosome rhythm is calculated at all CLOCK:BMAL1 peaks targeting rhythmic target genes (left), or only at peaks targeting rhythmic non core clock genes (removal of nucleosome signal at peaks targeting Cry2, Dbp, Rev-erbα, and Rev-erbβ for the Rinφ group, and of the peaks targeting Per1 and Per2 for the Ro/φ group. E: maximal and minimal nucleosome signal from the 6-time points rhythms for each of the CLOCK:BMAL1 transcriptional output groups. Groups with different letters are significantly different (2-way ANOVA; p < 0.05). (TIF) [file pgen.1007156.s007.tif]

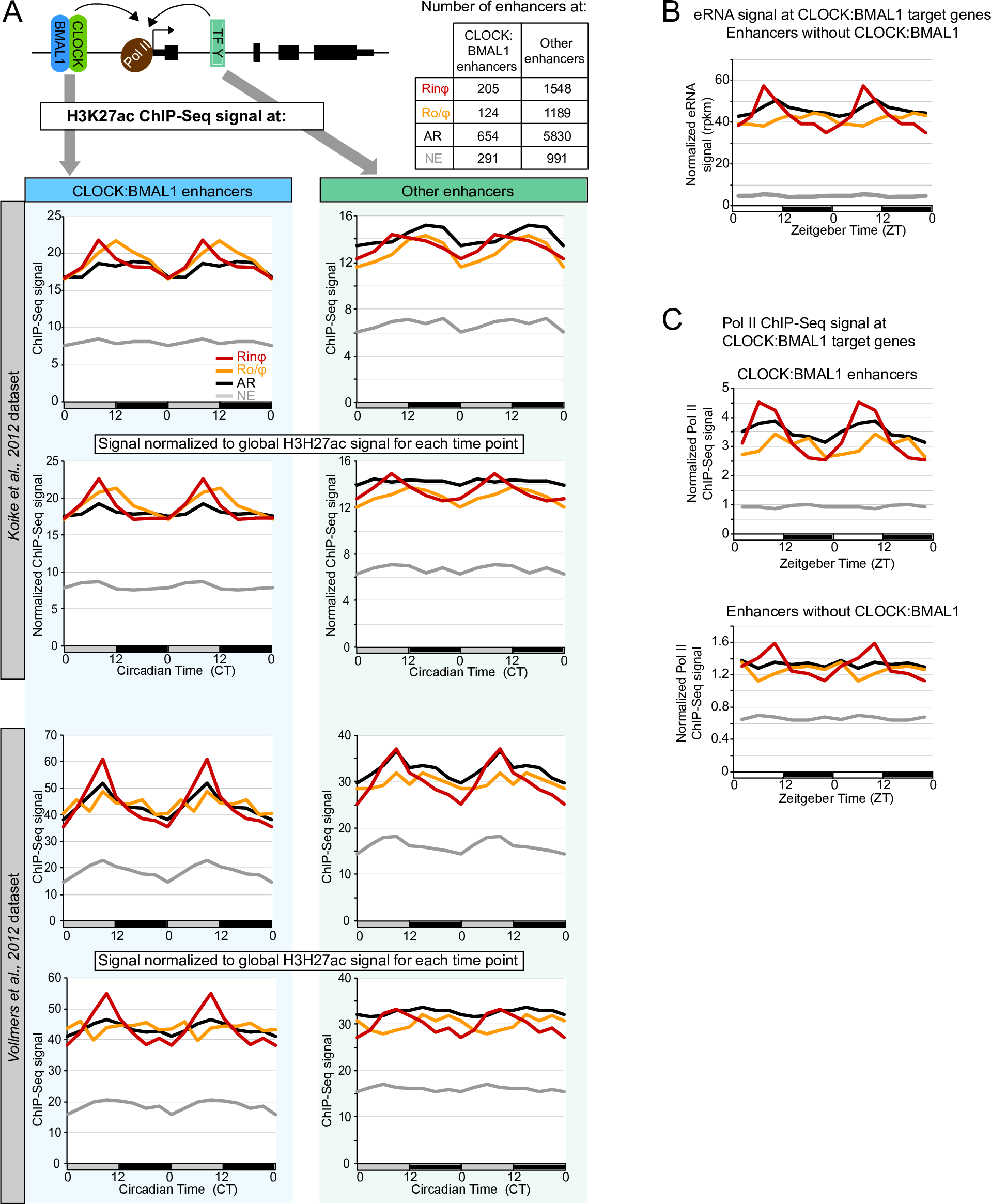

Supplement: S8 Fig — A. Circadian rhythm of H3K27ac ChIP-Seq signal in the mouse liver at CLOCK:BMAL1 DNA binding sites (Left; blue background) or at non-CLOCK:BMAL1 enhancers located in CLOCK:BMAL1 target genes (Right; green background) for each of the 4 CLOCK:BMAL1 transcriptional output groups: rhythmic-in-phase (Rinφ, red); rhythmic out-of-phase (Ro/φ, orange); arrhythmic (AR, black); and non expressed (NE, grey) target genes. Datasets were retrieved from Koike et al., 2012 (top) or Vollmers et al., 2012 (bottom) and re-analyzed (see methods section for more details). Values correspond to the ChIP-Seq signal median for each group, and were calculated for each CLOCK:BMAL1 peak as the average of reads/bp at CLOCK:BMAL1 DNA binding sites center ± 1 kb normalized to one million sequencing reads. For each dataset, H3K27ac ChIP-Seq signal was further normalized by mean normalization to account for the differences in ChIP-Seq efficiency between each sequencing sample (bottom graphs for each datasets). This normalization assumes that the overall genome-wide levels of H3K27ac are constant at any time in the mouse liver. To this end, we normalized H3K27ac ChIP-Seq signal for each peak to the averaged H3K27ac signal calculated at the top 40,000 DNase hypersensitive sites for each time point Graphs are double-plotted to improve visualization. B. Rhythm of enhancer RNA (eRNA) signal in the mouse liver at enhancers targeting a CLOCK:BMAL1 target gene and that do not harbor a CLOCK:BMAL1 DNA binding site (calculated at CLOCK:BMAL1 peak center ± 500 bp) for each of the 4 CLOCK:BMAL1 transcriptional output group. Datasets were retrieved from Fang et al., 2014 [36] and re-analyzed (see methods section for details). Values correspond to the eRNA signal median for each group. Each rhythm is double-plotted for better visualization. C. Rhythm of RNA Polymerase II ChIP-Seq signal in the mouse liver at CLOCK:BMAL1 enhancers (top) and enhancers targeting a CLOCK:BMAL1 target gene but without a CLOCK:BMAL1 D [file pgen.1007156.s008.tif]

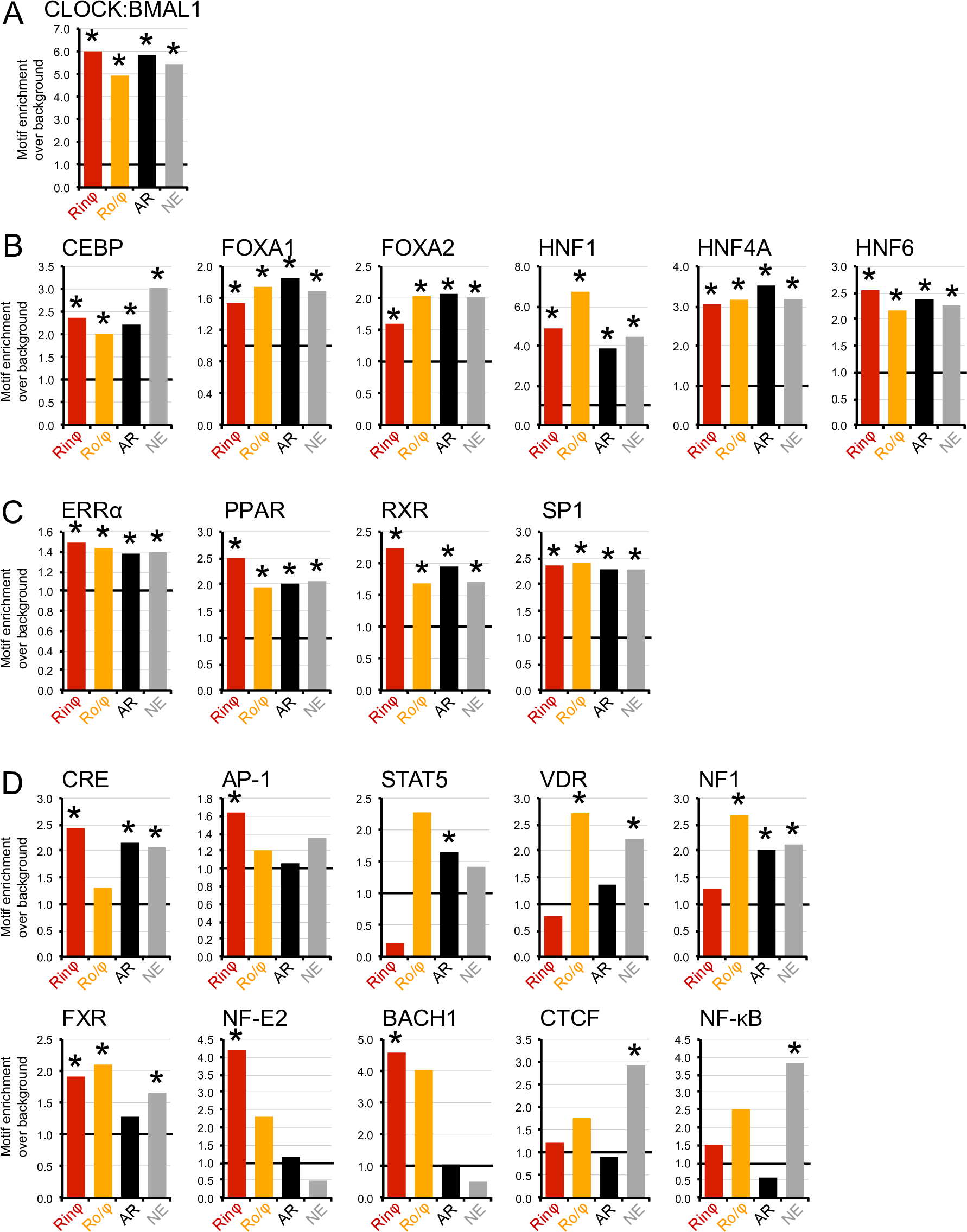

Supplement: S9 Fig — Enrichment for transcription factor DNA binding motifs (calculated using the HOMER software suite) at CLOCK:BMAL1 DNA binding sites for each of the four CLOCK:BMAL1 transcriptional output categories: rhythmic-in-phase (Rinφ, red); rhythmic out-of-phase (Ro/φ, orange); arrhythmic (AR, black); and non expressed (NE, grey) target genes. Enrichment is reported as the ratio between the calculated enrichment over the calculated background. * q < 0.05 (Benjamini-Hochberg procedure). Motif enrichment is shown for: CLOCK:BMAL1 (A); tissue-specific transcription factors (B); as well as ubiquitous transcription factors for which the motif is enriched for all 4 output groups (C) or specific group(s) (D). (TIF) [file pgen.1007156.s009.tif]

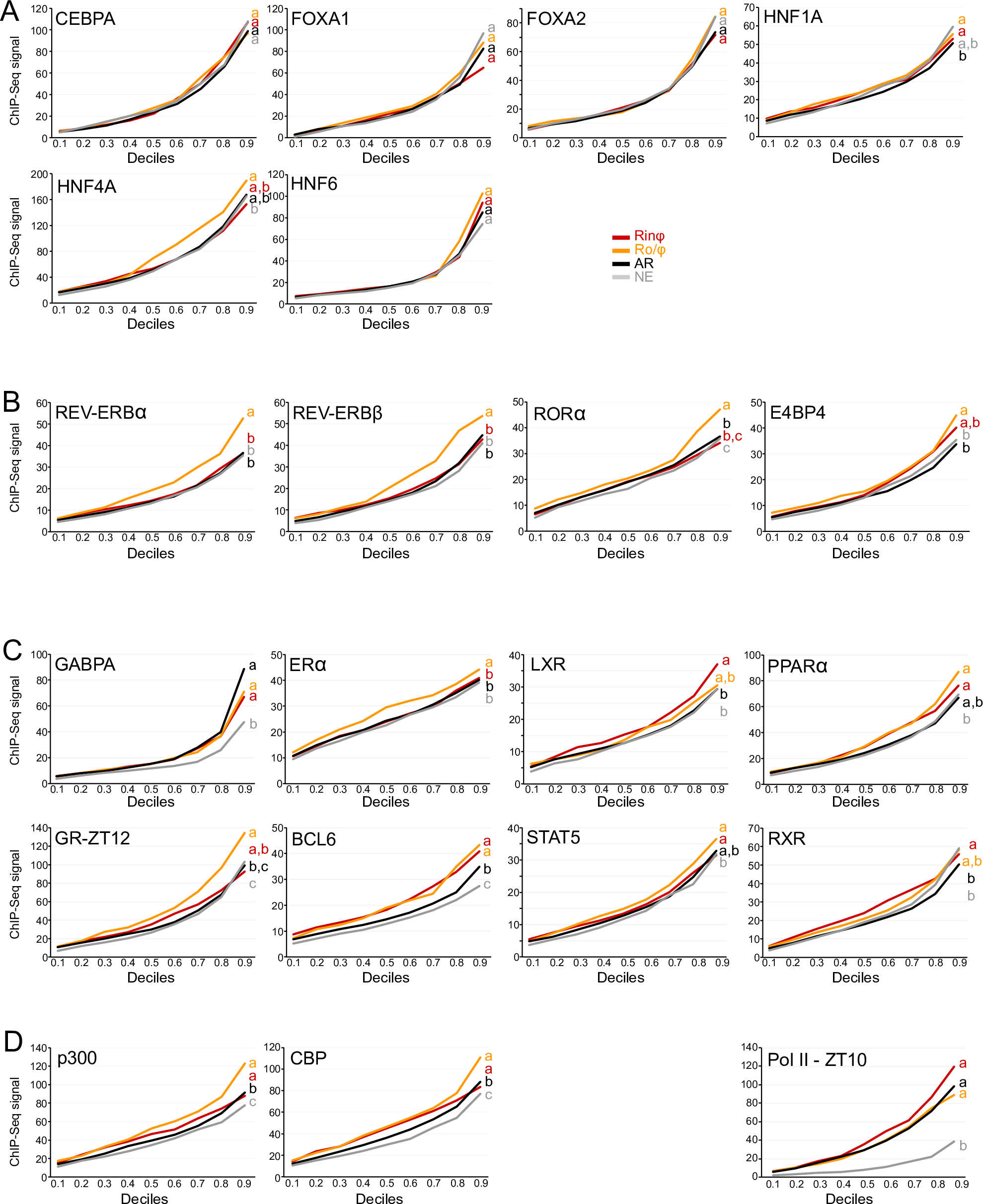

Supplement: S10 Fig — Mouse liver ChIP-Seq signal of tissue-specific transcription factors (A), ubiquitous transcription factors (B, C), and transcriptional co-activators / RNA Polymerase II at ZT10 (D) at CLOCK:BMAL1 DNA binding sites (peak center ± 250bp) for each of the transcriptional output categories. ChIP-Seq signal is represented for each output group based on its distribution (every decile). Groups with different letters are significantly different (Kruskal-Wallis test; p < 0.05). (TIF) [file pgen.1007156.s010.tif]

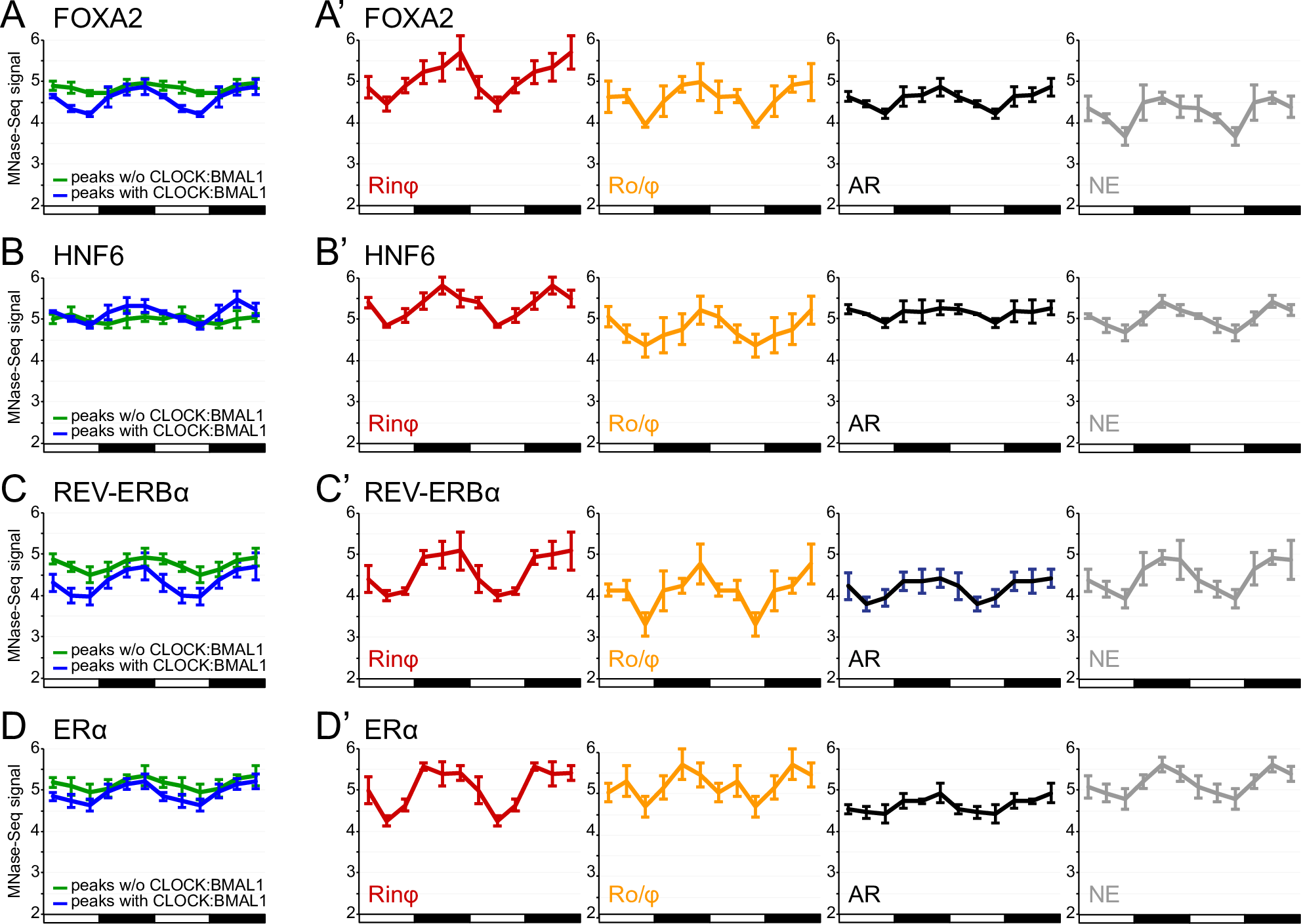

Supplement: S11 Fig — Nucleosome signal at four TF DNA binding sites was retrieved from mouse liver MNase-Seq datasets (Menet et al., 2014), which consists of 6 time points each separated by 4 hours with n = 4 mice for each time point. MNase-Seq data are displayed for two tissue-specific TFs: FOXA2 (A) and HNF6 (B), and two ubiquitous TFs: REV-ERBα (C) and ERα (D). Nucleosome signal was calculated at TF peak center ± 75 bp for each peak and at each TF ChIP-Seq peak coordinate, and is displayed as the average ± s.e.m of the signal (n = 4) calculated for each mouse. Each rhythm is double-plotted for better visualization. (Left, A-D) Nucleosome signal at TF ChIP-Seq peaks harboring a CLOCK:BMAL1 peak (blue), or at the top 10,000 TF ChIP-Seq peaks that do not harbor a CLOCK:BMAL1 peak (green). (Right, A’-D’) Nucleosome signal at TF ChIP-Seq peaks harboring a CLOCK:BMAL1 peak, and parsed based on CLOCK:BMAL1 transcriptional output: Rhythmic-in-phase (Rinφ, red), Rhythmic out-of-phase (Ro/φ, orange), arrhythmic (AR, black), and non expressed (NE, grey). (TIF) [file pgen.1007156.s011.tif]

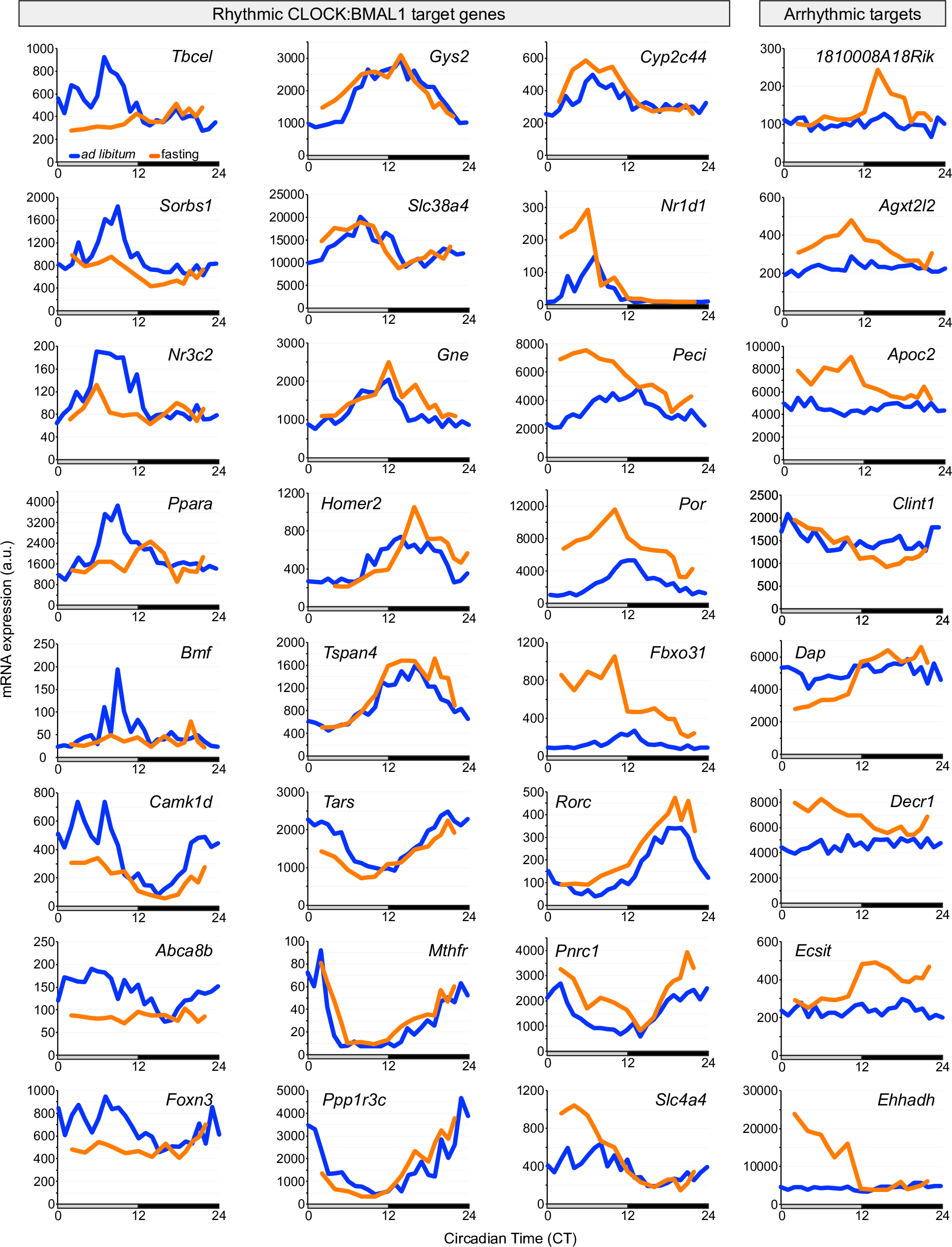

Supplement: S12 Fig — Rhythm of mRNA expression in the liver of mice fed ad libitum (blue) or fasted for at least 22 hours (orange). Data were retrieved from a public dataset (Vollmers et al., 2009). Mouse liver mRNA expression is displayed for CLOCK:BMAL1 target genes that are rhythmically expressed in the liver of mice fed ad libitum, and that exhibit under fasting condition a decrease in the rhythm amplitude (first column), no change (second column) or an increase in the rhythm amplitude (third column). Mouse liver mRNA expression is also displayed for CLOCK:BMAL1 target genes that are arrhythmically expressed in the liver of mice fed ad libitum, and that exhibit rhythmic expression under fasting condition. (TIF) [file pgen.1007156.s012.tif]

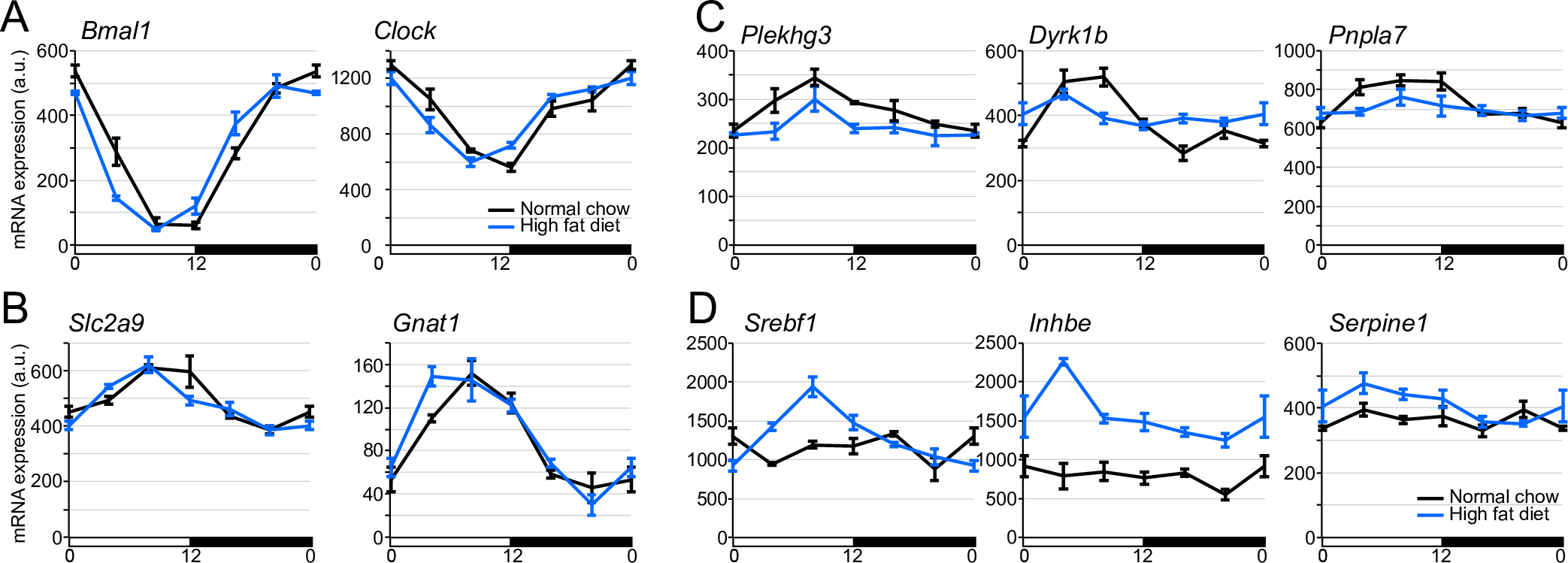

Supplement: S13 Fig — A-D. Six-time point rhythm of liver mRNA expression in mice fed with normal chow (black) or high fat diet (blue). Values were retrieved from a public dataset [57] and correspond to the average ± s.e.m. of 3 independent samples. Mouse liver mRNA expression is displayed for Clock and Bmal1 (A), and some CLOCK:BMAL1 target genes that are rhythmic under both normal chow and high fat diet (B); normal chow only (C); and high fat diet only (D). (TIF) [file pgen.1007156.s013.tif]

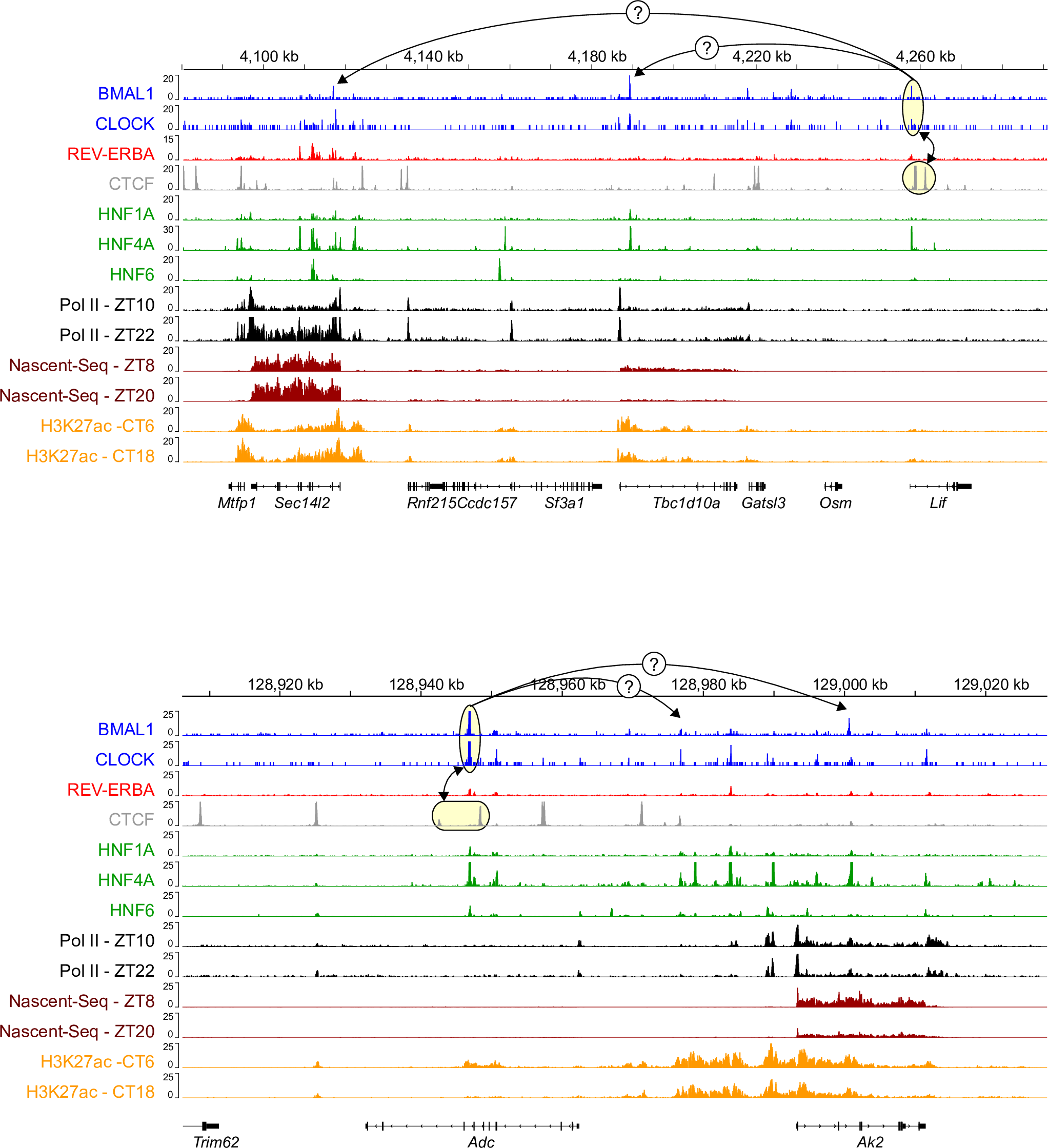

Supplement: S14 Fig — Analysis of a random set of genes not targeted by CLOCK:BMAL1 and transcribed similarly to the 4 CLOCK:BMAL1 transcriptional output groups (n = 125 genes for each group) was performed to determine the extent to which the findings reported in the manuscript are specific to CLOCK:BMAL1. The same criteria as those used for the characterization of CLOCK:BMAL1 transcriptional output were used, and groups are thus similarly referred as Rhythmic-in-phase (Rinφ, dark blue), Rhythmic out-of-phase (Ro/φ, light blue), arrhythmic (AR, black), and non expressed (NE, grey). Cis-regulatory regions targeting the randomly selected control genes are defined as DNase I hypersensitive sites (DHS) located within -10kb of the TSS to +1kb of the TTS (similarly to what was done for CLOCK:BMAL1 target genes). Analysis of these DHS suggests that many of our findings are specific to CLOCK:BMAL1 A. Heatmap displaying nascent RNA expression of the random set of genes and parsed based on the transcriptional output. Nascent-Seq signal was ordered based on the phase of nascent RNA oscillations for the in-phase and out-of-phase transcriptional cyclers. Ordering of arrhythmically transcribed genes is based on the peak time of maximal expression; the lack of a distinctive 24-hr rhythm profile of nascent RNA expression over the 48-hr time-scale is indicative of arrhythmic transcription. NE genes are not displayed due to the lack of expression. B. Average nascent RNA expression level for the 4 control groups. C-D. BMAL1 (C) and CLOCK (D) ChIP-Seq signal at DNase I hypersensitive sites (DHS) targeting the randomly selected control genes. ChIP-Seq signal for CLOCK:BMAL1 target genes is provided for comparison. E. Nucleosome rhythm at DHS targeting the randomly selected control genes (similar to Fig 3A–3D). F-H. H3K27ac, Pol II and eRNA expression at DHS targeting the randomly selected control genes (similar to Figs 3F, S8C and S3G, respectively). I-K. ts-TF (I), u-TF (J), and p300 and Pol II (K) ChIP-Seq [file pgen.1007156.s014.tif]

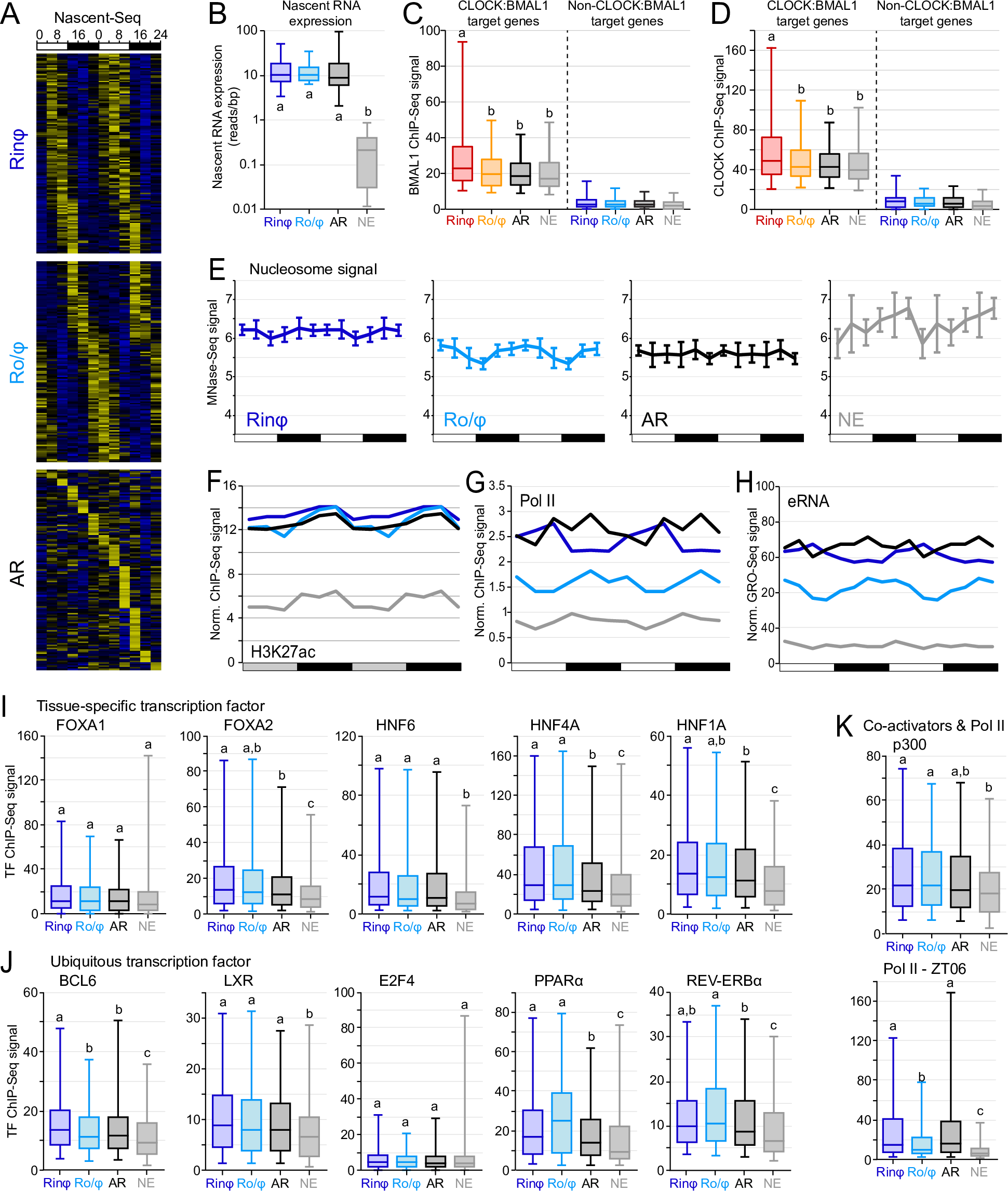

Supplement: S15 Fig — Visualization file of ChIP-Seq signal at two CLOCK:BMAL1 enhancers (yellow boxes) targeting non expressed genes (Top: Lif and Bottom: Adc), and exhibiting significant CTCF ChIP-Seq signal. Arrows with question marks indicate a potential CTCF-mediated long-range chromatin interaction that would enable CLOCK:BMAL1 to regulate the rhythmic transcription of genes located more than 50kb away from its enhancer. (TIF) [file pgen.1007156.s015.tif]
